# Identification of Penicillin Binding Protein 4 (PBP4) as a critical factor for *Staphylococcus aureus* bone invasion during osteomyelitis in mice

**DOI:** 10.1371/journal.ppat.1008988

**Published:** 2020-10-22

**Authors:** Elysia A. Masters, Karen L. de Mesy Bentley, Ann Lindley Gill, Stephanie P. Hao, Chad A. Galloway, Alec T. Salminen, Diamond R. Guy, James L. McGrath, Hani A. Awad, Steven R. Gill, Edward M. Schwarz

**Affiliations:** 1 Center for Musculoskeletal Research, University of Rochester Medical Center, Rochester, NY, United States of America; 2 Department of Biomedical Engineering, University of Rochester Medical Center, Rochester, NY, United States of America; 3 Department of Pathology and Laboratory Medicine, University of Rochester Medical Center, Rochester, NY, United States of America; 4 Department of Orthopaedics, University of Rochester Medical Center, Rochester, NY, United States of America; 5 Department of Microbiology and Immunology, University of Rochester Medical Center, Rochester, NY, United States of America; National Institutes of Health, UNITED STATES

## Abstract

*Staphylococcus aureus* infection of bone is challenging to treat because it colonizes the osteocyte lacuno-canalicular network (OLCN) of cortical bone. To elucidate factors involved in OLCN invasion and identify novel drug targets, we completed a hypothesis-driven screen of 24 *S*. *aureus* transposon insertion mutant strains for their ability to propagate through 0.5 μm-sized pores in the **Micro**fluidic **Si**licon **M**embrane **C**analicular **A**rrays (μSiM-CA), developed to model *S*. *aureus* invasion of the OLCN. This screen identified the uncanonical *S*. *aureus* transpeptidase, penicillin binding protein 4 (PBP4), as a necessary gene for *S*. *aureus* deformation and propagation through nanopores. *In vivo* studies revealed that Δpbp4 infected tibiae treated with vancomycin showed a significant 12-fold reduction in bacterial load compared to WT infected tibiae treated with vancomycin (p<0.05). Additionally, Δpbp4 infected tibiae displayed a remarkable decrease in pathogenic bone-loss at the implant site with and without vancomycin therapy. Most importantly, Δpbp4 *S*. *aureus* failed to invade and colonize the OLCN despite high bacterial loads on the implant and in adjacent tissues. Together, these results demonstrate that PBP4 is required for *S*. *aureus* colonization of the OLCN and suggest that inhibitors may be synergistic with standard of care antibiotics ineffective against bacteria within the OLCN.

## Author summary

*Staphylococcus aureus* is the most prevalent pathogen in osteomyelitis, and its infection of bone is difficult to cure. *S*. *aureus* colonization of the osteocyte lacuno-canalicular network (OLCN) of cortical bone has been identified as a novel pathogenetic mechanism in chronic osteomyelitis. To elucidate factors involved in OLCN invasion, we conducted an *in vitro* genetic screen that identified *pbp4* as a critical gene for *S*. *aureus* cell deformation and propagation through nanopores and demonstrated that PBP4 is critical for OLCN colonization in murine osteomyelitis. Thus, PBP4 inhibitors may be novel drugs to treat osteomyelitis in combination with standard of care antibiotics.

## Introduction

Osteomyelitis is a devastating disease caused by bacterial infection of the bone, for which treatment guidelines are suboptimal [1,2] and oftentimes require surgical intervention in addition to extended antimicrobial therapy [3]. *Staphylococcus aureus* is the most common pathogen isolated from chronic osteomyelitis [4], with 50% of prosthetic joint infections caused by hard-to-treat methicillin resistant *S*. *aureus* (MRSA) [2,5]. Despite advances in medical technology, the incidence of infection across all classes of orthopaedic subspecialties ranges from 0.1–30% [4] and rates of reinfection following revision surgery remain as high as 33% [6,7]. Further, the recurrence of *S*. *aureus* osteomyelitis following decades of quiescence and presumptive cure remains an important clinical problem [8–10]. Most recently, expert consensus has challenged the use of antibiotic-loaded bone cement (ALBC), which is a standard of care treatment for *S*. *aureus* osteomyelitis, despite the substantial lack of evidence to demonstrate clinical efficacy of ALBC [11]. These experts highlighted the great need to develop novel antibiotics that specifically target *S*. *aureus* infection of bone, which is considered very challenging to treat [11].

*S*. *aureus* persistence in chronic osteomyelitis can be attributed to a variety of immune evasion mechanisms specific to the bone microenvironment [12,13]. These mechanisms include *Staphylococcus* abscess communities (SACs) within the bone marrow and soft tissue [14–16], biofilm formation on necrotic tissue and implant hardware when present [17,18], and most notably, invasion and colonization of the immune-privileged osteocyte lacuno-canalicular network (OLCN) of cortical bone [19,20]. While extensive debridement and removal of all foreign bodies can address SACs and surface biofilms during revision surgery for infected orthopaedic implants, amputation remains the only theoretical treatment to remove *S*. *aureus* from OLCN of live bone. Thus, there is great interest in novel antibiotics that can address this unmet clinical need.

Systematic examination of infected murine and human bone by transmission electron microscopy (TEM) revealed that *S*. *aureus* is capable of deforming, invading and colonizing the submicron sized networks of canaliculi, connecting the lacunar spaces of osteocytes within cortical bone [19,20]. This invasion of the OLCN by *S*. *aureus* requires the non-motile cocci to deform into a rod-shaped bacterium at diameters less than half of its native size [20]. Descriptive TEM micrographs and *in vivo* metabolic labeling studies lead to the theory that *S*. *aureus* invades and colonizes canaliculi via haptotaxis, motility induced by bound ECM molecules [21], to orient the bacterium at the orifice, and durotaxis, motility induced by matrix stiffness [22], to extrude daughter cells into and throughout canaliculi via asymmetric binary fission. In support of this hypothesis, careful investigation of *S*. *aureus* cell division mechanics has revealed that daughter cells separate on the timescale of milliseconds [23], suggesting the involvement of strong mechanical forces imposed by the bacterial cell walls [24], which is capable of extruding a daughter cell into the canalicular space.

**Fig 1:**
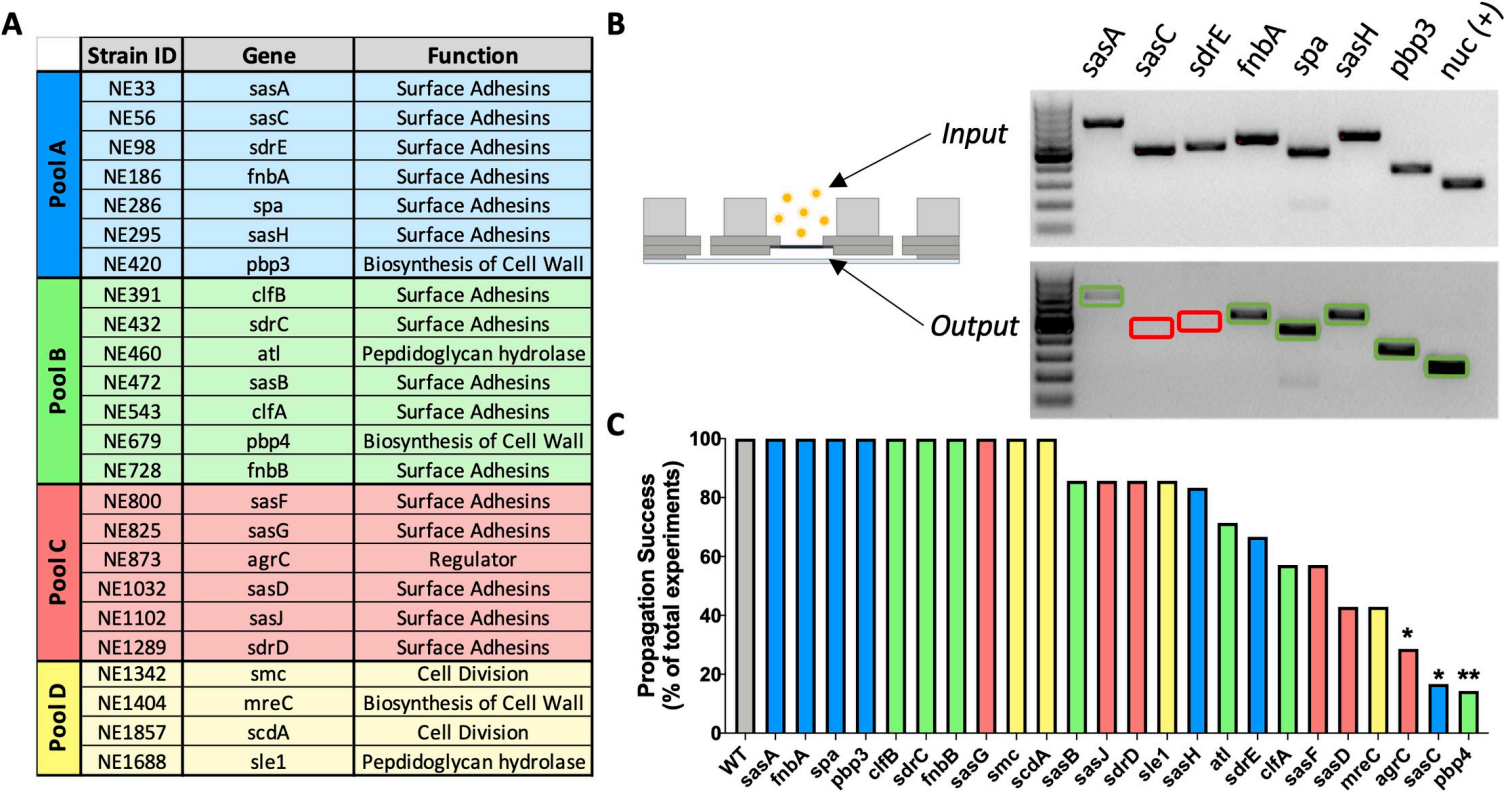
A hypothesis-driven *in vitro* genetic screen of *S*. *aureus* mutants to identify genes required for propagation through nanopores. Twenty-four USA300 *S*. *aureus* mutants from the Nebraska Transposon Mutant Library (NTML) were obtained for *in vitro* screening of genes hypothesized to be involved in OLCN invasion based on their known functions (A). Mutant strains were randomly pooled into four groups, and screened in the μSiM-CA system, where groups of 4–7 strains were cultured above the nanoporous membrane (input), and those that propagated through the membrane (output) were identified by PCR using strain specific primers (B). A representative image of the PCR products electrophoresed in an agarose gel is shown to illustrate equivocal input of the mutants from Pool A (top), and binary confirmation of strain presence in the output (bottom), with amplification of *nuc* as a positive control. Note the complete absence of *sasC* and *sdrE* PCR products in the output of this representative experiment (red boxes), while successful propagation by the other mutants is indicated by their PCR product (green boxes). The number of μSiM-CA propagation experiment where a strain successfully propagated through the nanoporous membrane was summed and plotted as a percentage of total experiments performed (B). WT USA300 is plotted as the positive control (grey bar) and mutant pools are indicated by colors matching panel A. Of the 24 mutants screened, only *agrC*, *sasC* and *pbp4* displayed a significantly decreased propagation efficiency compared to 100% propagation efficiency of WT USA300 *S*. *aureus* (C; *p<0.05, **p<0.01 vs. WT by Fisher’s exact test).

Currently, the genetic mechanisms regulating *S*. *aureus* deformation and propagation through the OLCN are poorly understood. Thus, elucidation of *S*. *aureus* genes associated with OLCN invasion represents a novel class of antibiotic targets for the prevention and treatment of chronic osteomyelitis. To this end, we developed and characterized the **Micro**fluidic–**Si**licon **M**embrane–**C**analicular **A**rray (μSiM-CA), which is an *in vitro* model designed to mimic the dimensions and rigidity of canaliculi using a silicon membrane with spatially pattered 0.5 μm pores [25]. Here we describe a hypothesis-driven genetic screen in which 24 *S*. *aureus* mutants with transposon disruptions in genes hypothesized to be involved in OLCN invasion, based on their known function, were assessed for their ability to propagate through the μSiM-CA (Fig 1A). Specifically, we hypothesize that *S*. *aureus* invade the OLCN through a process that involves cell deformation and asymmetrical binary fission into the canaliculus. Therefore, the genes screened in this study were selected based on their roles in cell division, cell wall biosynthesis and hydrolysis, synthesis of external adhesins and gene regulators. The hypothesis-driven screen identified penicillin binding protein 4 (PBP4) as a critical factor *S*. *aureus* cell deformation and invasion *in vitro*, which we validated in a murine model of implant-associated osteomyelitis. Thus, this work identifies PBP4 as a novel antibiotic target for therapies to prevention and treat chronic osteomyelitis.

## Results

To determine which candidate *S*. *aureus* genes are essential for submicron-scale invasion and colonization of the OLCN, we screened a library of transposon insertion mutants for their ability to propagate through the nanopores of the μSiM-CA. Screening of the mutant library was conducted in four discrete pools (Fig 1A), with binary confirmation of strain propagation by PCR (Fig 1B). The completed screen revealed that strains with mutations in *pbp4*, *sasC* and *agrC* have significantly decreased propagation success compared to WT USA300 (Fig 1C). The *pbp4* mutant strain (NE679) successfully propagated through nanopores, to the lower chamber, in only 1 of 7 replicate experiments. While *agrC* and *sasC* mutants showed similar significant deficiencies in nanopore propagation, *pbp4* was selected as the primary candidate gene for continued investigation in this work because of its role in cell wall biosynthesis. While not significant, mutant strains *mreC*, *sasD*, *sasF* and *clfA* showed decreased propagation success, p = 0.0699, 0.0699, 0.1923, and 0.4615, respectively.

**Fig 2:**
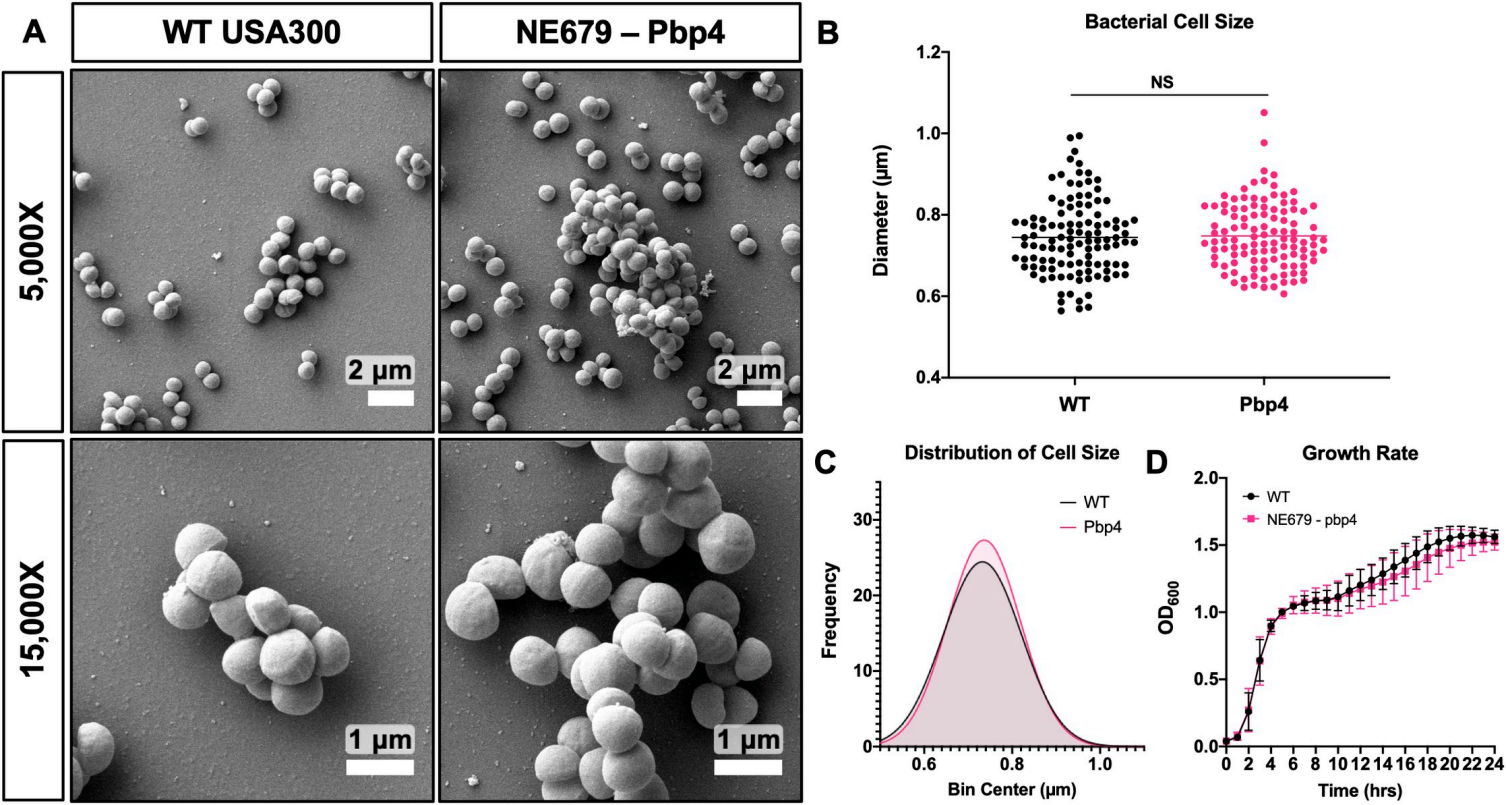
*Pbp4* deficient *S*. *aureus* displays normal morphology and growth characteristics *in vitro*. Cultures of WT USA300 *S*. *aureus* and the *pbp4* transposon mutant (NE679) were processed for SEM (n = 3 independent experiments), and representative images are shown to illustrate the absence of distinctive morphological differences between the two strains (A). Bacterial cell size was quantified as the maximum cell diameter from six SEM images, and the data for each bacterium with mean for the strain are presented (B; p = 0.7558, by one-way student’s T-test), with the distribution of cell diameters (C; p = 0.862,5 by Kolmogorov-Smirnov test for cumulative distributions). WT and NE679-Pbp4 grown in liquid culture and measured by optical density at 600 nm, hourly for 24 hrs, show statistically similar growth rate at all time points via two-way ANOVA with Sidak’s post-hoc for multiple comparisons (D, n = 3, data presented as mean +/- SD).

To eliminate potential confounding factors of altered cell morphology and impaired generation time leading to decreased propagation, mutant strains identified in the genetic screen were evaluated by SEM imaging and growth rate analysis. WT *S*. *aureus* and *pbp4* transposon mutant, NE679, showed no remarkable differences in cell morphology (Fig 2A). Furthermore, *pbp4* mutation did not affect bacterial cell diameter, bacterial cell size distribution, or growth rate, compared to WT (Fig 2B–2D). Together, these results demonstrate that the decreased nanopore propagation phenotype observed with the NE679 strain is specific to cell deformation and invasion, and not a result of altered cell morphology or proliferation.

Additional mutant strains identified in the genetic screen, *agrC* and *sasC*, were also evaluated for changes in cell morphology and growth rate (S1 Fig). SEM results confirmed an expected cell clumping phenotype of the *agrC* mutant [25], as well as a slightly increased mean cell diameter and hindered growth during stationary phase. *SasC* mutant cells were statistically similar to WT in cell size and in growth rate.

**Fig 3:**
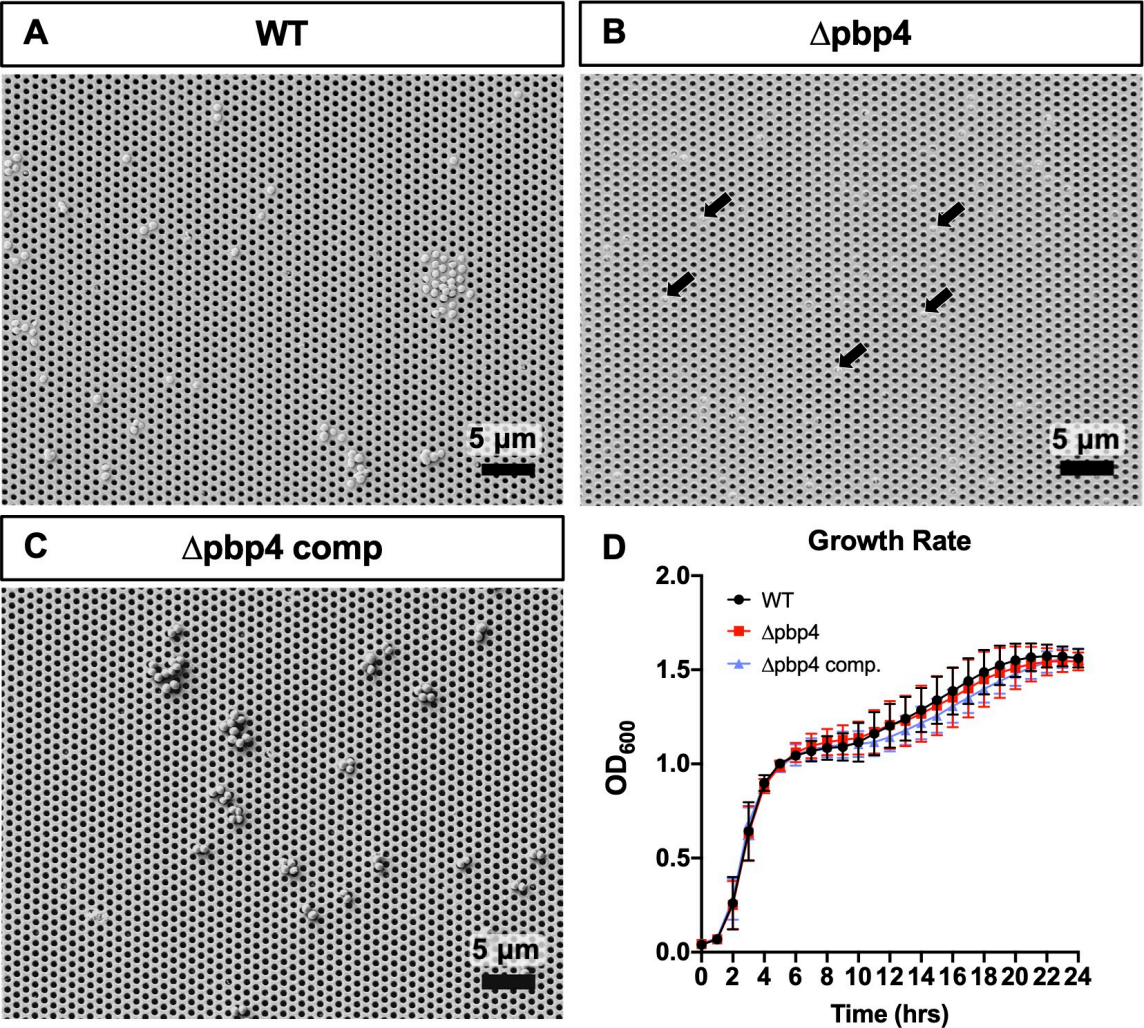
Isogenic mutant and complementation confirmation of *pbp4* deficient phenotypes *in vitro*. Pure cultures of isogenic strains wild-type USA300 (WT), USA300 *pbp4* deletion mutant (Δpbp4) and its complement strain (Δpbp4 comp) were assayed for propagation potential in the μSiM-CA device (n = 4 independent experiments), and representative SEM images of the bottom surfaces of the membrane are shown at 2000X (A-C). WT bacteria readily propagate through the 0.5 μm pores following 6 hours of incubation (A). Note that Δpbp4 bacteria cultured on top of the membrane can be observed occupying pore openings from the bottom side (arrows in B), but no Δpbp4 bacteria were found in the bottom chamber. In contrast, Δpbp4 comp. bacteria were readily observed propagating through the nanopores and on the bottom surface of the membrane, similar to WT (C). WT, Δpbp4 and Δpbp4 complement grown in liquid culture and measured by optical density at 600nm, hourly for 24 hrs, show similar growth rate at all time points via two-way ANOVA with Sidak’s post-hoc for multiple comparisons (D, n = 3, data presented as mean +/- SD).

To validate the results of the genetic screen, which used pools of transposon insertion mutants, monoculture μSiM-CA nanopore propagation experiments were performed. It is known that WT *S*. *aureus* readily propagates through the 0.5 μm pores within 6 hours (Fig 3A) [25]. In contrast, a *pbp4* deletion mutant of *S*. *aureus*, USA300 Δpbp4, was incapable of propagating through the nanopores (Fig 3B). Lack of Δpbp4 propagation was first noted when media aspirated from the bottom well of the μSiM-CA showed zero bacterial growth after 24 hours of incubation, and further validated by performing SEM imaging of the bottom of the membrane. Notably, bacterial cells can be seen on top of the membrane occupying pore openings, but do not pass through the pores. The inability for USA300 Δpbp4 to propagate through nanopores was restored by *pbp4* complementation on a plasmid (Fig 3C). Importantly, the growth rate of WT, Δpbp4 and Δpbp4 complement USA300 were all equivalent, with no significant difference in growth at any time points (Fig 3D). Thus, we conclude that PBP4 is required for *S*. *aureus* propagation through nanopores that are similar to canaliculi in size and rigidity.

μSiM-CA validation studies of the *agrC* and *sasC* transposon mutant monocultures were also performed. μSiM-CA studies revealed that the *agrC* mutant does indeed propagate through nanopores (S2 Fig). Thus, the false-positive identification of *agrC* in the genetic screen was likely due to its competitive disadvantage as a result of its larger cell size and hindered growth in stationary phase versus the other mutants assayed in Pool C. μSiM-CA studies with *sasC* transposon mutant monoculture showed that this strain failed to propagate through nanopores, similar to the *pbp4* mutant (S2 Fig). Thus, future *in vitro* and *in vivo* studies with *sasC* isogenic mutants are warranted to confirm its potential role in invasion and propagation in canaliculi.

**Fig 4:**
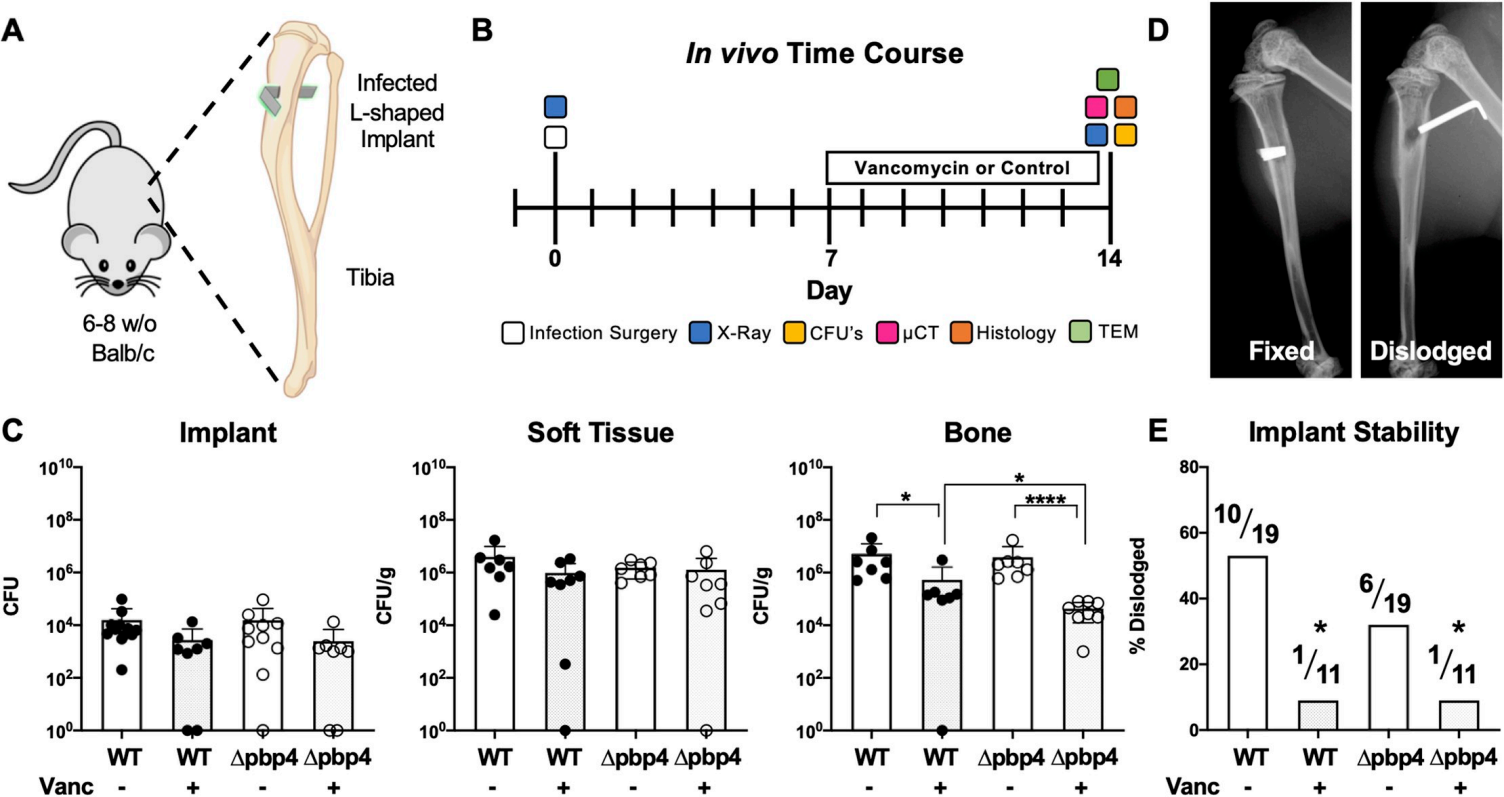
*Pbp4* deletion and vancomycin therapy reduce bacterial load in bone and prevent septic implant loosening during *S*. *aureus* implant-associated osteomyelitis. L-shaped wires contaminated with WT or Δpbp4 USA300 were surgically implanted through the tibia of mice as indicated in the schematic (A). One week later, mice received vancomycin (110 mg/kg i.p. twice daily) or placebo treatment prior to sacrifice on day 14 (B). Following sacrifice, tibiae were harvested and processed for CFU quantification or for μCT, histology and TEM. CFUs were quantified from the implant, soft tissue and bone, and the data for each mouse with the mean +/- SD for each group is presented (C). No differences in CFUs between any groups were found on the implant or in adjacent soft tissue. Significance differences in CFUs in bone between groups are shown (*p<0.05, ****p<0.0001, by one-way ANOVA, with Tukey’s post-hoc for multiple comparisons). Additionally, X-rays were obtained at the time of sacrifice to determine if the implant remained fixed (left) or was dislodged (right) from the tibia (D). Consistent with the significant reduction in CFU in bone of vancomycin treated bone, this antibiotic treatment also significantly prevented dislodgment of the implants (D, *p<0.05 vs WT, by Fisher’s exact test).

Following the identification and confirmation of *pbp4* as a candidate gene involved in cell deformation and propagation through nanopores *in vitro*, *pbp4* deletion mutant was evaluated for invasion and propagation in canaliculi *in vivo*. A murine model of implant-associated osteomyelitis was performed, where L-shaped stainless-steel wire pins were contaminated with WT USA300, USA300 Δpbp4, or maintained sterile, and implanted into the tibiae of 6–8 week-old, female Balb/C mice (Fig 4A). Seven days later, the infected mice received vancomycin or PBS placebo treatment, prior to sacrifice on day 14, when the animals were X-ray imaged and infected tibiae were harvested and processed for colony forming unit (CFU) quantification, μCT, histology and TEM analyses (Fig 4B). Prior to beginning *in vivo* studies, the vancomycin MIC was determined to be equal for both WT and Δpbp4 strains of USA300 (S3 Fig).

**Fig 5:**
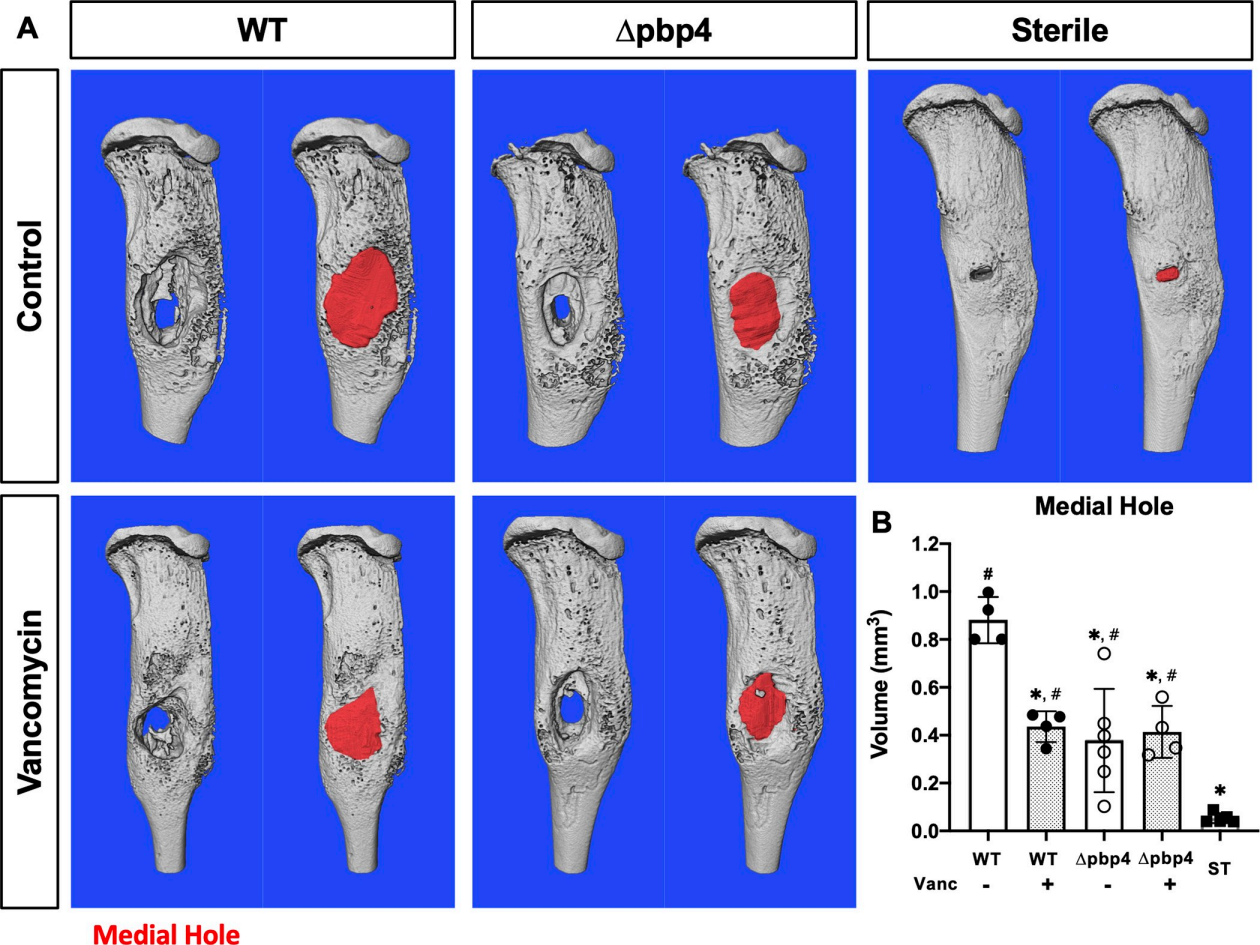
*Pbp4* deletion and vancomycin therapy reduce osteolysis around *S*. *aureus* infected implants. Sterile or *S*. *aureus* contaminated tibial implants, WT or Δpbp4 USA300, were implanted into mice (n ≥ 4), and vancomycin or placebo treatments were administered as previously described. Implanted tibiae were harvested on day 14 post-infection for μCT analyses. The μCT DICOM scans were reconstructed using Amira and medial hole volume was identified though the depth of the tibial cortex by manual segmentation and interpolated between slices. Representative 3D reconstructions of the μCT scans for all experimental groups are shown without (left) and with (right) medial hole void filled (red regions in A). The medial hole volume for each tibia is presented with mean +/- SD for each group (B). Statistical significance compared to WT is indicated by * (p<0.05), and significance compared to ST indicated by # (p<0.05) by one-way ANOVA, with Tukey’s post-hoc for multiple comparisons.

CFU quantification demonstrated that there were no significant differences in bacterial load on the implant or in infected soft tissue across all groups (Fig 4C). In bone, however, vancomycin treatment resulted in a significant decrease in bacterial load for both WT and Δpbp4 infections. While *pbp4* deletion alone had no effect on bone bacterial load compared to WT within placebo treated groups, vancomycin treatment was significantly more effective against Δpbp4 infection compared to WT infection treated with vancomycin. Radiographic assessment of septic implant loosening revealed that vancomycin therapy significantly reduced implant dislodgement (Fig 4D and 4E). Additionally, within the placebo treated groups, *pbp4* deletion also reduced implant dislodgment compared WT. Consistently, μCT assessment of the medial hole volume revealed that both vancomycin treatment and *pbp4* deletion reduce osteolysis around the infected implants (Fig 5).

**Fig 6:**
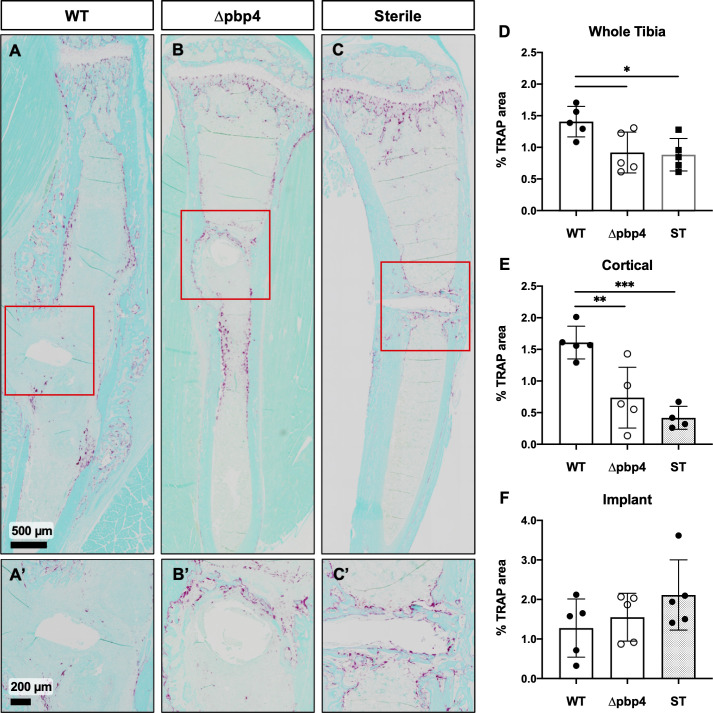
Infection by Δpbp4 *S*. *aureus* induces significantly less osteoclast activation in cortical bone. Histologic sections from sterile and infected tibiae were stained for TRAP (red/purple) with fast green counterstain. Representative images of TRAP stained WT- and Δpbp4-infected and sterile implant control tibiae are shown at x1 (A, B, C) and x4 at the implant site (A’, B’, C’). % TRAP stained area was quantified within the whole tibia, cortical bone regions and implant sites (D-F, n = 5–6 presented as mean +/- SD). Significance was evaluated by one-way ANOVA with Tukey’s post-hoc for multiple comparisons, *p < 0.05, **p < 0.01, ***p < 0.001.

To elucidate differences in bone resorption among WT- and Δpbp4-infected tibiae, histologic sections were stained for tartrate-resistant acid phosphatase (TRAP) positive osteoclasts and % TRAP area was quantified using Visiopharm image analysis (Fig 6). Quantification of TRAP staining showed significantly increased osteoclast presence within the whole tibia and within the cortical bone region of WT-infected tibiae compared to Δpbp4-infected and sterile tibiae (Fig 6D and 6E). Osteoclast quantification at the implant site showed no statistical difference among any groups (Fig 6F). Note TRAP staining of osteoclasts at the implant site is proportional to bone resorption as well as the amount of new woven bone, visible in the sterile pin control (Fig 6C’).

**Fig 7:**
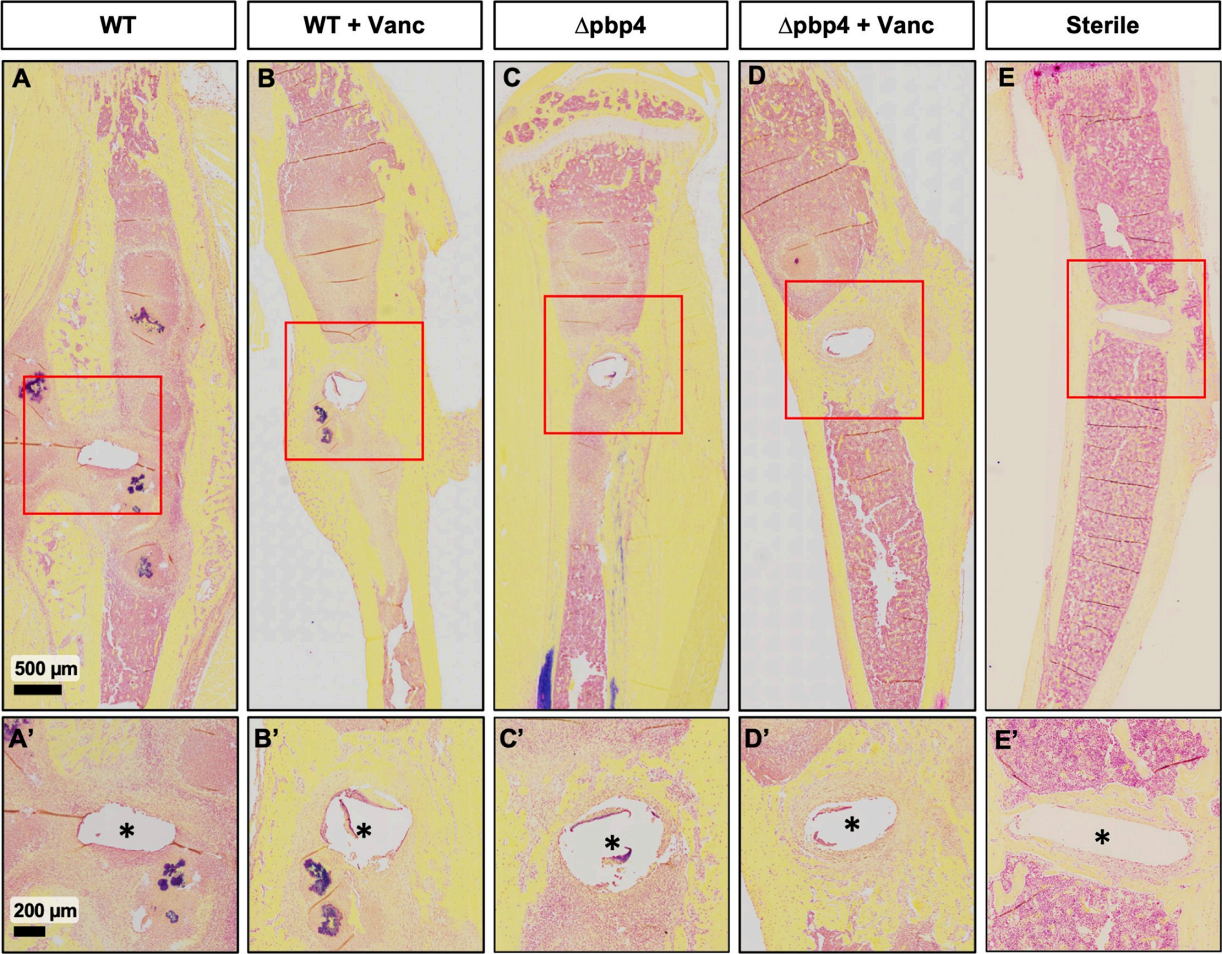
Histologic confirmation of reduced Gram-positive bacteria and increased bone formation around the implant in tibiae infected with *pbp4* deficient *S*. *aureus*. Infected tibiae described in Fig 5 were processed for Brown-Brenn stained histology, and tissue sections from each group are shown at 1x and 4x magnification. Note the abundant Gram-positive bacteria (dark purple) within *Staphylococcus* abscess communities (SACs) and granulation tissue around the implant site (*) in WT USA300 infected tibiae (A, A’, B, B’), which were present in all samples from these groups. While these features were also present in Δpbp4 infected tibiae, *pbp4* deletion resulted in a marked increase of bone formation around the implant site compared to WT (C, C’, D, D’). Sterile implant control tibiae show noticeably greater osseous integration compared to all infection groups (E, E’).

Next, Brown-Brenn modified Gram stain was used to identify Gram-positive *S*. *aureus* (Fig 7). All WT-infected tibiae show abundant Gram-positive stained SACs throughout the bone marrow cavity and adjacent to the implant site (Fig 7A), and robust granulation tissue formation (Fig 7A’). Consistent with prior reports [26,27], vancomycin treatment of WT infected tibiae failed to eradicate the SACs (Fig 7B and 7B’). Remarkably, Δpbp4-infected tibiae show evidence of improved osseous integration as implicated by apparent new woven bone at the size of the implant, with less osteolysis compared to untreated, WT-infected tibiae (Fig 7C and 7C’). As expected, vancomycin treatment of Δpbp4 infected tibiae show robust bone formation surrounding the implant (Fig 7D and 7D’). Nonetheless, Δpbp4 *S*. *aureus* continued to form robust SACs with and without vancomycin treatment (S4 Fig), and no significant differences in total abscess numbers across all groups were observed (S4 Fig).

**Fig 8:**
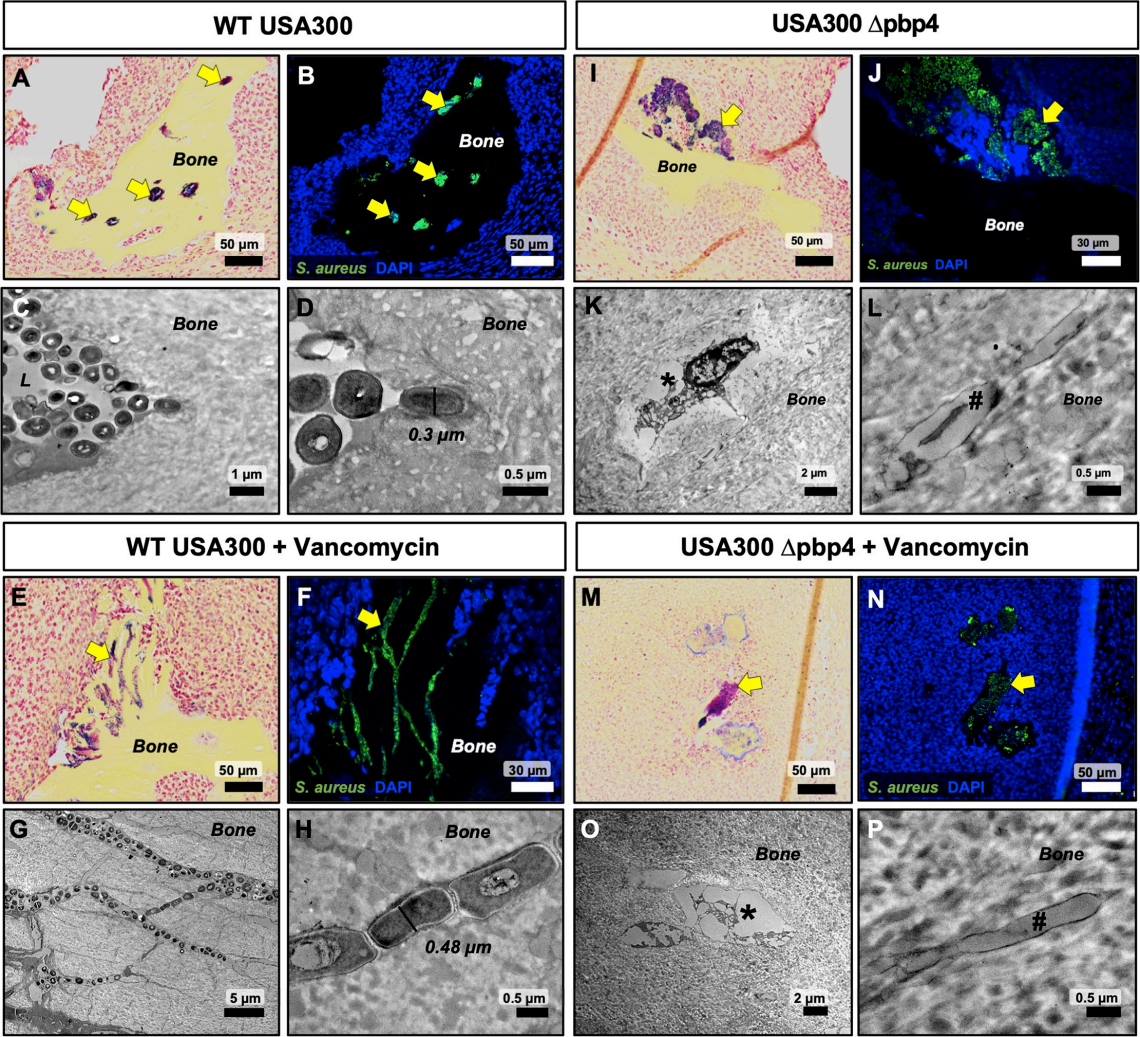
Lack of OLCN invasion by Δpbp4 *S*. *aureus* in the murine model of implant-associated osteomyelitis. Brown-Brenn stained histology sections were used to identify necrotic bone fragments containing Gram positive bacteria in tibiae infected with WT and Δpbp4 USA300, and adjacent tissue sections (n = 3 tibiae per group) were used for: 1) immunofluorescent histochemistry (IHC) labeling with a polyclonal anti-*S*. *aureus* antibody to identify all *S*. *aureus* in the section, and 2) ultrastructural analysis via TEM “pop-off” method to formally interrogate OLCN invasion. Assessment of placebo treated tibiae infected with WT USA300 confirmed the specificity of the IHC (corresponding yellow arrows in A pointing to Gram positive bacteria in A and green immunofluorescence in B), and TEM evidence of MRSA colonization of vacant osteocyte lacunae (C) and submicron bacteria invading adjacent canaliculi (D). Assessment of vancomycin treated tibiae infected with WT USA300 confirmed that this antibiotic treatment has no detectable effects on MRSA invasion of OLCN (E-H). In contrast, USA300 Δpbp4 infected tibiae only contained bacteria attached to the bone surface and not in submicron channels (I-P). Careful interrogation of these infected bone fragments revealed no evidence of OLCN invasion or submicron bacteria in a vacant lacuna (*) and canaliculus (#) (K-L & O-P). Additionally, no remarkable effects of vancomycin treatment on USA300 Δpbp4 infected bone were observed (I-L vs. M-P).

Histological sections of infected tibiae were further interrogated by immunohistochemistry (IHC) using an anti-*S*. *aureus* antibody and blinded ultrastructural analysis by TEM “pop-off” method to formally evaluate OLCN invasion (Fig 8). Histological assessment of WT-infected tibiae revealed extensive colonization of the osteocyte-lacunar spaces within necrotic bone fragments (Fig 8A and 8B). TEM micrographs also confirm OLCN colonization (Fig 8C and 8D). Remarkably, vancomycin treatment had no detectable effects on WT USA300 invasion and colonization of the OLCN (Fig 8E–8H). In contrast, assessment of tibiae infected with Δpbp4 *S*. *aureus* showed bacterial colonization restricted to the surface of necrotic bone, regardless of treatment (Fig 8I–8P). TEM interrogation of Δpbp4-infected bone fragments failed to identify any evidence of bacterial invasion or colonization of OLCN in all samples, which was evidenced by empty osteocyte-lacunae and canaliculi in placebo and treatment groups (Fig 8K–8L & 8O–8P). All biological replicates are shown in S5 Fig, S6 Fig, S7 Fig and S8 Fig.

## Discussion

The finding that *S*. *aureus* is capable of invading and colonizing the OLCN of cortical bone provides a deeper explanation as to why chronic osteomyelitis is considered to be very challenging to treat, and necessitates the development of better treatments [19,20]. As this novel pathogenic process is inconsistent with the established dogma of *S*. *aureus* as a non-motile coccus ~1 μm in diameter, the elucidation of the genes involved in invasion and colonization of the OLCN will provide greater insights into this deadly human pathogen.

Previously we have validated the utility of the μSiM-CA platform to distinguish the phenotype of *S*. *aureus* strains based on propagation through nanopores [25]. Here we expand on this work by screening a target mutant library, assembled from NTML transposon insertion mutant strains, of *S*. *aureus* genes hypothesized to contribute to OLCN invasion through changes in cell wall structure, cell division and cell shape. Interestingly, the genetic screen identified three genes with very different functions to be important for *S*. *aureus* cell shape deformation and propagation through nanopores. The genes identified include: 1) *pbp4*, encoding a non-essential, low molecular weight PBP of *S*. *aureus* [28] known specifically for its role in secondary cross-linking of peptidoglycan [29,30]; 2) *sasC*, encoding a relatively uncharacterized cell surface associated protein suggested to be involved in cell aggregation and biofilm formation [31], and 3) *agrC*, encoding the transmembrane receptor histidine kinase of the *S*. *aureus* accessory gene regulator (Agr), known as the primary modulator of virulence factors during infection [32,33]. While not significant, cell division protein *mreC* showed decreased nanopore propagation success, suggesting the importance of cell division in *S*. *aureus* invasion of submicron-sized environments. Interestingly, three genes encoding surface adhesins in addition to *sasC* (*sasD*, *sasF* and *clfA*) trended toward decreased propagation through nanopores suggesting that adherence may be an important step for invasion.

Characterization of mutant strain cell morphology, cell size and growth rate, followed by validation studies in the μSiM-CA with pure mutant cultures allowed for elimination of possibly confounding factors for observed differences in nanopore propagation. Importantly, we found that *pbp4* transposon mutant cells were equivalent to WT in size and in growth rate indicating that its propagation deficiency is not caused by alternative mechanisms. Further, USA300 *pbp4* deletion and complement strains confirm that *S*. *aureus* deformation and propagation through nanopores is dependent on *pbp4* expression.

Characterization of additional mutant strains identified in this work, *sasC* and *agrC*, revealed that *sasC* remains a possible gene involved in nanopore propagation and OLCN invasion and therefore requires continued research. On the other hand, *agrC* was likely identified as a false positive in the genetic screen due to its aggregation phenotype and increased cell diameter. Indeed, foundational work using the μSiM-CA found that the accessory gene regulator (*agr*) of *S*. *aureus* is not required for bacterial propagation through nanopores *in vitro*, nor invasion of the OLCN *in vivo* [25].

Following the *in vitro* genetic screen and subsequent validation studies, *pbp4* was selected as the primary candidate gene for investigation in *S*. *aureus* OLCN invasion. PBP4 is one of four genome encoded penicillin binding proteins (PBP) of *S*. *aureus*. PBPs, named for their affinity to the β-lactam class of antibiotics, are responsible for the final steps of cell wall biosynthesis [34]. The primary function of PBPs is to catalyze the crosslinking of peptidoglycan chains by a pentaglycine bridge [35,36]. PBP4 is specifically known for its role in the high degrees of peptidoglycan crosslinking readily observed in *S*. *aureus* [29,30].

*In vivo* infection of murine tibiae with WT or Δpbp4 *S*. *aureus* revealed that the bacterial load of implant hardware and soft tissue remains unchanged by treatment with vancomycin or *pbp4* deletion. This result is not surprising for two reasons. First, delayed antibiotic treatment is known to be ineffective against established biofilms and abscesses, as *S*. *aureus* is less susceptible to vancomycin in a sessile state compared to planktonic [37,38]. Second, we do not expect that the deletion of a nonessential transpeptidase would impact the strain’s ability to colonize the implant or soft tissue. Notably, *pbp4* deletion does not reduce the bacterial load within bone compared to WT. However, *pbp4* deletion increases the strains susceptibility to vancomycin therapy in bone tissue. We expect that both WT and Δpbp4 colonize bone tissue with similar overall bacterial burden, but in different spatial cavities. Specifically, WT *S*. *aureus* is capable of colonizing the surface of bone as well as within OLCN of bone, while Δpbp4 mutant *S*. *aureus* exclusively colonizes the surface of bone. As a result, the Δpbp4 mutant is more susceptible to killing by systemic vancomycin.

Historically, it has been reported that *pbp4* mutation may be involved in resistance to glycopeptides like vancomycin [39]. This result is not particularly surprising given that vancomycin is effective by inhibiting cell wall biosynthesis, however vancomycin specifically binds the C-terminal D-Ala-D-Ala peptide of peptidoglycan precursors prior to transpeptidation by PBPs. Importantly, the MIC values of WT and Δpbp4 were determined to be equivalent in this work. Additionally, more recent reports have described unchanged susceptibility to vancomycin following *pbp4* deletion [40], together confirming that vancomycin resistance as a result of *pbp4* deletion is not a concern.

μCT analysis of infected and sterile tibiae revealed that *pbp4* deletion resulted in decreased bone loss at the implant site compared to WT, despite equivalent bacterial loads measured by CFU quantification. This reduction in bone loss was comparable to the effect of vancomycin treated WT infections, while vancomycin treatment of Δpbp4 infections had no effect. These results, echoed by improved implant stability associated with *pbp4* deletion and vancomycin treatment, indicate that *pbp4* deletion may reduce osteolysis by a mechanism similar to that of vancomycin therapy, given that both affect changes in cell-wall crosslinking.

Quantification of TRAP^+^ osteoclasts within cortical bone regions of infected and sterile tibiae suggests that *pbp4* deletion results in decreased osteoclast-mediated bone resorption in *S*. *aureus* infection. We posit that *pbp4* deficient *S*. *aureus*, incapable of invading the submicron canaliculi, do not activate the production of receptor activator of NFκB ligand (RANKL) from osteocytes resulting in diminished osteoclast-mediated bone resorption adjacent to the infection [41–44]. Alternatively, *S*. *aureus* peptidoglycan can directly stimulate the production of proinflammatory cytokines by the activation of TLR-2 [45] and hydrolysis of *S*. *aureus* peptidoglycan cross-links reduces the release of proinflammatory cytokines [46]. Thus, *S*. *aureus* deficient of key peptidoglycan cross-linker, PBP4, may result in diminished induction of the host proinflammatory response and ultimately diminished bone resorption.

Histological evidence of newly woven bone complements this finding by suggesting *pbp4* deletion and vancomycin therapy both allow for increased bone formation at the implant site, despite the presence of SACs in all infection groups. Therefore, future studies further characterizing the impact of *pbp4* expression on bone homeostasis should be performed.

An important finding of this work is the ultrastructural analysis of infected bone by TEM. Through repeated and blinded interrogation of infected bone by histological and TEM methods, OLCN invasion was not observed in tibiae infected with *S*. *aureus* deficient in *pbp4*. While it is impossible to definitively prove a negative result, altogether, the results of this study suggest for the first time that expression of *pbp4* may be required for the deep *S*. *aureus* invasion of cortical bone during osteomyelitis.

Currently, the direct mechanism of PBP4 involvement in OLCN invasion remains to be elucidated. We theorize that the dynamic regulation of cell wall biosynthesis by *S*. *aureus* PBPs during cell division may play an important role. It is understood that peptidoglycan synthesis determines bacterial shapes and provides the driving force for cell membrane and cell wall invagination [47]. Therefore, PBPs can be implicated in *S*. *aureus* deformation and invasion in the OLCN.

Important work by Loskill *et al*. found that *pbp4* deletion reduces the elastic modulus (stiffness) of the cell wall by 2-4-fold in MRSA strains using atomic force microscopy (AFM). These results suggest that cell wall stiffness may play an important role in OLCN invasion where, in contrast to initial theories, cell wall rigidity may be necessary for the generation of force required for daughter cell propagation within the OLCN. Complementary to PBP4 mediated peptidoglycan crosslinking, loss of autolysin activity such as glucosaminidase (*gmd*) results in increased peptidoglycan chain length and a stiffer cell wall [48]. In agreement of this, individual mutation of autolysins *atl* and *sle* does not significantly diminish bacterial propagation through nanopores.

Finally, the factors regulating *pbp4* transcription are poorly understood. It can be assumed that *pbp4* expression is tightly controlled, as it is dynamically tuned in response to local antibiotic concentrations [49]. Recently, studies have shown that PBP4 may play an important role in *S*. *aureus* resistance to β-lactam antibiotics [40,50–53]. Therefore, other factors contributing to the regulation of *pbp4* activity should be investigated further, including wall teichoic acids, shown to act as spatial and temporal regulations of PBP4 mediated peptidoglycan crosslinking [54], and PBP2, which has been shown to interact with PBP4 during cell wall synthesis [55].

In conclusion, this work presents for the first time a systematic approach to uncover the genetic mechanism(s) of *S*. *aureus* invasion of the OLCN. By this method, we have determined that *pbp4* is necessary for the submicron invasion of canaliculi. Additionally, deletion of *pbp4* results in a unique infection phenotype, with decreased pathogenic bone loss despite the presence of high bacterial loads. Together, this work confirms that the continued research of *pbp4* regulation and its role in deep bone invasion remains a priority. Ultimately, the development of a *pbp4*-specific small molecule inhibitor represents a potential novel antimicrobial therapy that could be used in combination with bacteriocidic compounds for the prevention of *S*. *aureus* OLCN invasion in osteomyelitis.

## Materials and methods

### Strains and growth conditions

*S*. *aureus* USA300, USA300 JE2 and their derivative mutant strains and primers used in this work are described in S1 Table and S2 Table, respectively. *S*. *aureus* strains were grown on tryptic soy agar (TSA) plates or in tryptic soy broth (TSB) at 37°C with shaking unless stated otherwise. A literature review identified 24 candidate *S*. *aureus* genes, which we hypothesized to be involved in OLCN invasion. A target library of these 24-transposon insertion mutant strains was assembled for screening in the μSiM-CA (Fig 1A). All 24 transposon mutant strains screened were provided by the Network on Antimicrobial Resistance in *Staphylococcus aureus* (NARSA) for distribution by BEI Resources, NIAID, NIH: Nebraska Transposon Mutant Library (NTML) Genetic Toolbox. All NTML mutants are created in the genetic background of prototypic *S*. *aureus* strain USA300 JE2. The *S*. *aureus* USA300 *pbp4*-null strain (USA300 Δpbp4) was constructed by allelic replacement using *Escherichia coli-Staphylococcus aureus* shuttle vector pWedge, as previously described [56]. Deletion was confirmed by PCR amplification and sequencing of the chromosomal region flanking *pbp4* in USA300. *Pbp4* complement was created by ligating the full-length deleted gene into pCN40 as previously described [57].

### μSiM-CA genetic screening

The μSiM-CA platform was developed to model *S*. *aureus* propagation through submicron geometry that mimics the canalicular network of cortical bone [25]. Briefly, this system features a 400 nm thick silicon nitride membrane with an array of 500 nm-sized pores fabricated by SiMPore Inc. (West Henrietta, NY, USA). High-throughput production of μSiM-CA was achieved by ALine Inc (Rancho Dominguez, CA). using laser cutting and lamination of acrylic, PET and COP layers bonded with pressure sensitive adhesives (PSAs), as previously described [25]. The resulting device contains defined top and bottom wells connected only through the nanoporous membrane.

A target mutant library of the 24 candidate genes was compiled from the Nebraska Transposon Mutant Library (NTML) and split into four discrete pools, A-D, for rapid screening in the μSiM-CA *in vitro* model for nanopore propagation. Pools were assigned randomly, simply based on numerical order of their strain ID. Bacterial cultures were prepared by growing overnight in TSB culture media and subcultured to mid-exponential phase growth. The μSiM-CA device was loaded by adding 10 μL of sterile tryptic soy broth (TSB) to the basal chamber of the device via the side inlet channels, and 80 μL of a mutant pool (containing 4–7 strains, denoted A-D) or pure *S*. *aureus* culture to the apical chamber above the nanoporous membrane.

*S*. *aureus* strains were incubated in the top chamber of the μSiM-CA at 37°C for 6 hrs. Following incubation in the μSiM-CA, apical (input) and basal (output) media was aspirated and out-grown overnight to expand the resultant bacterial populations. gDNA was isolated from the input and output out-grown cultures and mutant strains present in these cultures were identified by PCR using strain-specific primers. A gene-specific primer was used in combination with a transposon specific primer (either forward or backward) to identify each mutant strain, while concurrently verifying correct transposon location (S1 Table). Amplicons were electrophoresed in an agarose gel and imaged for binary confirmation of strain identity within the input and output cultures. Amplification of mutant strains in the bottom chamber (output) of the μSiM-CA following incubation represent genes that are not necessary for nanopore propagation (bands outlined green), whereas strains that do not appear in bottom chamber represent genes that are necessary for nanopore propagation (region outlined red) (Fig 1B). After 7 replicate experiments, the number of experiments a mutant strain successfully propagated to the bottom chamber (output) was divided by the total number of replicates to calculate propagation success.

### Growth rate measurements

*S*. *aureus* cultures were prepared by growing overnight, then subcultured the following day. Each strain of *S*. *aureus* was grown in a 96 well plate, at 37°C with shaking in a spectrophotometer and growth rate was evaluated by measuring optical density at 600 nm every hour from 0 to 24 hrs.

### Scanning electron microscopy (SEM) imaging

SEM imaging of the μSiM-CA was performed as previously described [25]. Briefly, the μSiM-CA system was fixed with 2.5% glutaraldehyde/4% paraformaldehyde in 0.1 M cacodylate buffer overnight. The bottom COP layer of the μSiM-CA device was peeled off to expose the underside of the membrane and post-fixed in buffered 1% osmium tetroxide, dehydrated in a graded series ethanol to 100% and critical point dried in a Tousimis CPD (Rockville, MD). Samples were mounted with the underside of the membrane exposed for imaging, to visualize bacteria which had propagated through the 0.5 μm nanopores. Finally, the membranes were sputter coated with gold and imaged using a Zeiss Arugia Field Emission SEM (Pleasanton, CA) for qualitative assessment of bacterial propagation.

Similarly, SEM was used to characterize bacterial cell morphology. Briefly, *S*. *aureus* cultures were grown overnight, then subcultured and seeded onto poly-L-lysine coated glass coverslips placed in a 24 well plates and statically incubated for 6 hours. Following incubation, bacterial media was aspirated from wells and coverslips were washed twice with phosphate buffered saline (PBS) before fixing. Cells were fixed, post-fixed, dehydrated and dried as described above. Samples were sputter coated with gold and imaged using a Zeiss Auriga Field Emission SEM for assessment of cell morphology. ImageJ, specifically Fiji [58], was used to measure the maximum cell diameter across 6 separate SEM images per cell type, where approximately 20 cells were measured in each image.

### Murine model for implant-associated osteomyelitis

All animal studies were performed in accordance with protocols approved by the University Committee on Animal Resources at the University of Rochester Medical Center and in accordance with the Animal Welfare Act. 6-week-old, female Balb/C mice were purchased from Jackson Laboratories (Bar Harbor, ME) and were acclimated for 1 week prior to surgery. Mice were housed five per cage in two-way housing on a 12-h light/dark cycle. Animal surgeries were performed as previously described [59,60]. Briefly, a flat stainless-steel wire with a cross-section of 0.2 mm x 0.5 mm (MicroDyne Technologies, Plainville, CT) was cut at 4 mm in length and bent into an L-shaped implant. Mice were anesthetized prior to surgery with xylazine (12 mg/kg) and ketamine (130 mg/kg) and were administered preoperative slow-release buprenorphine. The stainless-steel pins were first sterilized, then inoculated with an overnight culture of either WT USA300 or USA300 Δpbp4 for 20 minutes (approximately 5.0 x 10^5^ CFU/mL). The right hind-limb was shaved and washed with 70% ethanol then a 5 mm incision was created on the medial surface of the tibia. Next, the tibia was drilled with 30- and 26- gauge needles before carefully inserting the infected pin through the tibia. Finally, the muscle and skin were closed, and day 0 X-ray images were acquired to confirm proper pin placement (LX-60 X-Ray Cabinet, Faxitron Bioptics LLC; Tucson, AZ). From day 7 to day 14 post-infection select groups received 110 mg/kg systemic vancomycin, administered twice daily subcutaneously based on methods described by Caston *et al*. [61]. On day 14 post-infection, mice were sacrificed, and X-ray images were obtained to evaluate post-infection pin placement. Tibia, trans-tibial implant and soft tissue were harvested and placed in sterile PBS on ice for immediate CFU quantification or in neutral buffered formalin (NBF) for subsequent μCT imaging, followed by histology and TEM.

### CFU quantification

Infected tibia, transtibial implant and adjacent soft tissue were harvested and placed in sterile PBS on ice. Infected tibia and soft tissue were homogenized in 3 mL PBS in a 50 mL conical using an IKA T-10 handheld homogenizer (Wilmington, NC). Transtibial implants were sonicated in 1 mL sterile PBS for 2 min at 35 kHz (VWR Intl.; Radnor PA) to dislodge adhered bacteria and then vortexed. Tissue homogenate fluid and implant sonicate fluid were serially diluted in PBS and plated on TSA. Plates were incubated overnight, and resultant colonies were counted. Infected tibia and soft tissue were weighed prior to homogenizing and CFU’s were ultimately normalized to tissue mass.

### μCT imaging and analysis

Infected tibias were fixed in 10% NBF for 3 days at room temperature with associated soft tissue and implant left intact. Following fixation, samples were rinsed in PBS and distilled water, then gross soft tissue was dissected and transtibial implant was removed. Infected tibias were imaged *ex vivo* by micro-computed tomography (μCT) in a VivaCT 40 (Scanco Medical; Bassersdorf, Switzerland). Tibias were scanned with a 10.5 μm isotropic voxel size, using an integration time of 300 ms, energy of 55 kV, and intensity of 145 μA. Resultant DICOM files were used to create a 3D reconstruction of bone tissue using Amira software (FEI Visualization Sciences Group; Burlington, MA). Bone tissue was first binarized and reconstructed by thresholding. Medial hole volume quantification was performed by manual segmentation of the void area and interpolating through the depth of the tibial cortex.

### Histologic staining and analysis

Following fixation and μCT imaging, samples were placed in 14% EDTA tetrasodium for 7 days of decalcification. Samples were then paraffin processed and embedded transversely with the medial side of the tibia facing downwards. 5 μm transverse sections were cut and mounted on glass slides. Slides were deparaffinized and stained with Brown-Brenn modified Gram stain to visualize Gram positive bacteria. Brown-Brenn stain results in Gram positive organisms stained dark purple, cell nuclei stain pink and connective tissue stained yellow. Slides were digitized using a VS120 Virtual Slide Microscope (Olympus, Waltham, MA). *S*. *aureus* abscess communities (SAC’s) were quantified and averaged across 3 histological levels, for 4 biological replicates by manually counting SAC’s visualized in Olympus OlyVIA software.

TRAP staining was performed to visualize TRAP+ osteoclasts. TRAP stain results in TRAP+ osteoclasts stained red/purple with a blue/green tissue background. Slides were digitized using a VS120 Virtual Slide Microscope (Olympus, Waltham, MA). % TRAP area was quantified using an Analysis Protocol Package (APP) in Visiopharm (v.2019.07; Hoersholm, Denmark) within standardized regions of the interest (whole tibia, cortical bone or implant site). The APP utilizes colorimetric histomorphometry to detect TRAP staining (red/purple), fast-green counterstain (blue/green), and background (white) in order to accurately segment TRAP^+^ area for quantification. TRAP quantification was blinded.

### Immunofluorescence

Immunofluorescent staining was performed for detection of *S*. *aureus* in infected bone. Following deparaffinization, slides were incubated in sodium citrate solution at 95°C for 2 hours for antigen retrieval. Next, tissue was blocked in at room temperature in 5% normal goat serum (NGS) in 0.3% Triton-X100 TBS for 40 mins. Slides were incubated with polyclonal antibody for *S*. *aureus* (1:100, Invitrogen, Cat#: PA1-7246) in 5% NGS in 0.3% Triton-X100 TBS at 4°C overnight. Next, anti-rabbit FITC conjugated secondary antibody (1:400, Invitrogen, Cat#: A-11008) in 5% NGS in 0.3% Triton-X100 TBS was added to sections for 1 hr at room temperature. Finally, sections were counterstained with nuclear stain DAPI and mounted with ProLong Gold Antifade Mountant (Life Technologies, Eugene, OR). Stain specificity was validated by incubating with the secondary antibody only. Slides were either imaged using a VS120 Virtual Slide Microscope (Olympus, Waltham, MA) for abscess imaging or via confocal laser scanning microscopy (CLSM) for sequestra imaging. CLSM was performed using an inverted Olympus FV 1000 micrscope using a 60x oil immersion objective with 0.5 μm slices. Z-stack images were processed using ImageJ to create max-intensity z-projections.

### TEM “Pop-off” technique and imaging

Regions of interest within serially sectioned paraffin blocks of infected tibia samples, adjacent to Brown-Brenn stained sections, were processed for transmission electron microscopy using the “pop-off” technique, as previously described [62]. Briefly, slides were deparaffininzed in 3 changes xylene and then rehydrated through a graded series of ethanol to dH_2_0. Rehydrated sections were then post fixed in buffered 1% OsO_4_ for 20 minutes at room temperature. Slides were washed, dehydrated in a graded series ethanol to 100%, infiltrated for 1 hour with a 1:1 mixture of 100% ethanol and Spurr resin and embedded overnight in 100% resin. Regions of interest were polymerized in 100% Spurr resin under an inverted BEEM capsule for 24 hours at 65°C. Capsules were “popped off” slides by dipping 3–4 times in liquid nitrogen. Thin sections were cut at ~70 nm and placed onto formvar carbon coated nickel slot grids for imaging using a Hitachi 7650 TEM (Pleasanton, CA). Note that original formalin fixation of bone tissue, subsequent paraffin processing and embedding, and finally “popoff” for TEM, resulted in sub-optimal ultrastructural preservation. As a result, empty canaliculi often appeared as collapsed structures making imaging of non-infected bone tissue challenging. TEM imaging was performed for 3 biological replicates of all four groups, WT, WT + Vanc, Δpbp4 and Δpbp4 + Vanc. TEM imaging was blinded to sample group assignment. Representative images from all replicates are included in S5 Fig, S6 Fig, S7 Fig and S8 Fig, along with representative images of the adjacent Brown-Brenn and IHC sections.

### Minimum inhibitory concentration studies

The minimum inhibitory concentration (MIC) of vancomycin was measured for both the WT USA300 and USA300 Δpbp4 strains. In short, vancomycin was serially diluted from 256 to 0 μg/ml in TSB media in 96-well plate, and wells were inoculated with ~ 1.0 x 10^5^ CFU/ml of either WT USA300 or USA300 Δpbp4 and incubated overnight at 37°C, with shaking at 225 rpm. The MIC was defined as the lowest vancomycin concentration where there is no bacterial growth, evaluated by optical density measured at 600 nm. MIC was measured for 3 technical replicates and 3 biological replicates per strain.

### Statistical analyses

Fisher’s exact test was used for comparison of nominal data to a control group, including the genetic screen propagation success (%), and evaluation of implant stability. Unpaired t-test was used when two groups were compared, including cell diameter measurement. Kolmogorov-Smirov test was used to assess differences in cumulative cell size distributions. Two-way analysis of variance (ANOVA) with Sidak’s post-hoc for multiple comparisons was used to compare multiple variations such as differences in growth rate over time or MIC measurements. One-way ANOVA, with Tukey’s post-hoc for multiple comparisons was used for grouped data such as CFU’s and tibial hole volume. For statistical analysis, CFU data were log transformed to achieve normal distributions. All statistics were analyzed using GraphPad Prism.

## Supporting information

S1 Fig Mutant strain characterization studies with other NTML mutants identified in the μSiM-CA screen. Cultures of NE873-AgrC and NE56-SasC were assessed by SEM (A) and optical density at 600 nm (B) as described in Fig 2. The SEM results confirmed the predicted cell clumping phenotype of the *agrC* mutant, and also demonstrated that these bacteria are slightly larger than WT cells (B, *p<0.05, by one-way ANOVA with Tukey’s post-hoc for multiple comparisons). Interestingly, their growth rate is significantly hindered during stationary phase from 5–21 hours of incubation (D, two-way ANOVA with Sidak’s post-hoc for multiple comparisons). Taken together, the identification of NE873-AgrC in the genetic screen may be a false-positive because of its increased cell size and hindered growth. In contrast, SEM revealed that *sasC* mutants are similar to WT in size (B, C) and growth rate (D).
(TIFF)

S2 Fig μSiM-CA validation of pure bacterial cultures of AgrC and SasC NTML mutants. Cultures of NE873-AgrC and NE56-SasC were evaluated for nanopore propagation using methods described in Fig 3. Representative SEM micrographs (n = 4 independent experiments) confirm *agrC* mutant propagation through 0.5 μm nanopores (A, B), thereby confirming that this mutant strain was identified as a false-positive in the pooled genetic screen. In contrast, representative SEM micrographs (n = 4 independent experiments) of the *sasC* transposon mutant confirm that it is incapable of propagation through the nanopores (C, D).
(TIFF)

S3 Fig WT and Δpbp4 USA300 display similar MIC to vancomycin in vitro. Liquid cultures (n = 4) of WT and Δpbp4 USA300 were grown in the indicated concentration of vancomycin overnight at 37°C with shaking, and the optical densities of the cultures at 600 nm are presented as the mean +/- SD. No differences between strains were observed at any concentration (two-way ANOVA, with Sidaks’s post-hoc for multiple comparisons). The resultant MIC of both strains, simply measured by OD_600_, is approximately 8 μg/mL.
(TIFF)

S4 Fig WT and Δpbp4 SACs in placebo and vancomycin treated tibiae. Parallel histology sections of tibiae from the four infection groups were processed for Brown-Brenn staining and anti-*S*. *aureus* IHC as described in Fig 7, and representative micrographs of SACs in the tibial marrow space are shown at 20x. Note the overlapping Gram stained bacteria (purple) with the immunostaining (green). All infection groups show robust SAC formation, where *S*. *aureus* are located at the center of an abscess initially surrounded by a fibrous pseudo-capsule shown yellow in BB stain and as a black ring in IF staining (yellow arrow). Next, surrounding the fibrous pseudo-capsule is a ring of live and dead immune cells (red arrow), followed by living immune cells unable to penetrate the abscess structure (white arrow). Abscesses per tibia were quantified by averaging abscess number over 3 histological levels for 4 biological replicates (B). While there appears to be a trend toward decreased abscess number with vancomycin treatment and Δpbp4, no significant differences between groups were found (one-way ANOVA with Tukey’s post-hoc for multiple comparisons).
(TIFF)

S5 Fig Representative images of WT USA300 infected bone. Infected tibiae were processed for histology and sections were stained with Brown-Brenn (A, B, H, H, M, N), anti-*S*. *aureus* IHC (C, D, I, J, O, P) and processed for TEM “pop-off” (E, F, K, L, Q, R). TEM was imaging was blinded to sample group assignment. Representative images from all 3 biological replicates from this infection group are shown.
(TIFF)

S6 Fig Representative images of WT USA300 infected bone treated with vancomycin. Infected tibiae were processed for histology and sections were stained with Brown-Brenn (A, B, H, H, M, N), anti-*S*. *aureus* IHC (C, D, I, J, O, P) and processed for TEM “pop-off” (E, F, K, L, Q, R). TEM was imaging was blinded to sample group assignment. Representative images from all 3 biological replicates from this infection group are shown.
(TIFF)

S7 Fig Representative images of USA300 Δpbp4 infected bone. Infected tibiae were processed for histology and sections were stained with Brown-Brenn (A, B, H, H, M, N), anti-*S*. *aureus* IHC (C, D, I, J, O, P) and processed for TEM “pop-off” (E, F, K, L, Q, R). TEM was imaging was blinded to sample group assignment. Representative images from all 3 biological replicates from this infection group are shown.
(TIFF)

S8 Fig Representative images of USA300 Δpbp4 infected bone treated with vancomycin. Infected tibiae were processed for histology and sections were stained with Brown-Brenn (A, B, H, H, M, N), anti-*S*. *aureus* IHC (C, D, I, J, O, P) and processed for TEM “pop-off” (E, F, K, L, Q, R). TEM was imaging was blinded to sample group assignment. Representative images from all 3 biological replicates from this infection group are shown.
(TIFF)

S1 Table Strains used in this study. References: (1) Fey PD, et al. 2013. MBio 4:e00537-12. (2) Diep BA, et al. 2006. The Lancet 367:731–739.
(TIFF)

S2 Table Primers used in this study. (TIFF)
