# Supplementary figures and images for: Identification of Penicillin Binding Protein 4 (PBP4) as a critical factor for Staphylococcus aureus bone invasion during osteomyelitis in mice
Source: PLoS Pathog. 2020 Oct 22;16(10):e1008988. doi: 10.1371/journal.ppat.1008988 (PMC7608983; doi:10.1371/journal.ppat.1008988)

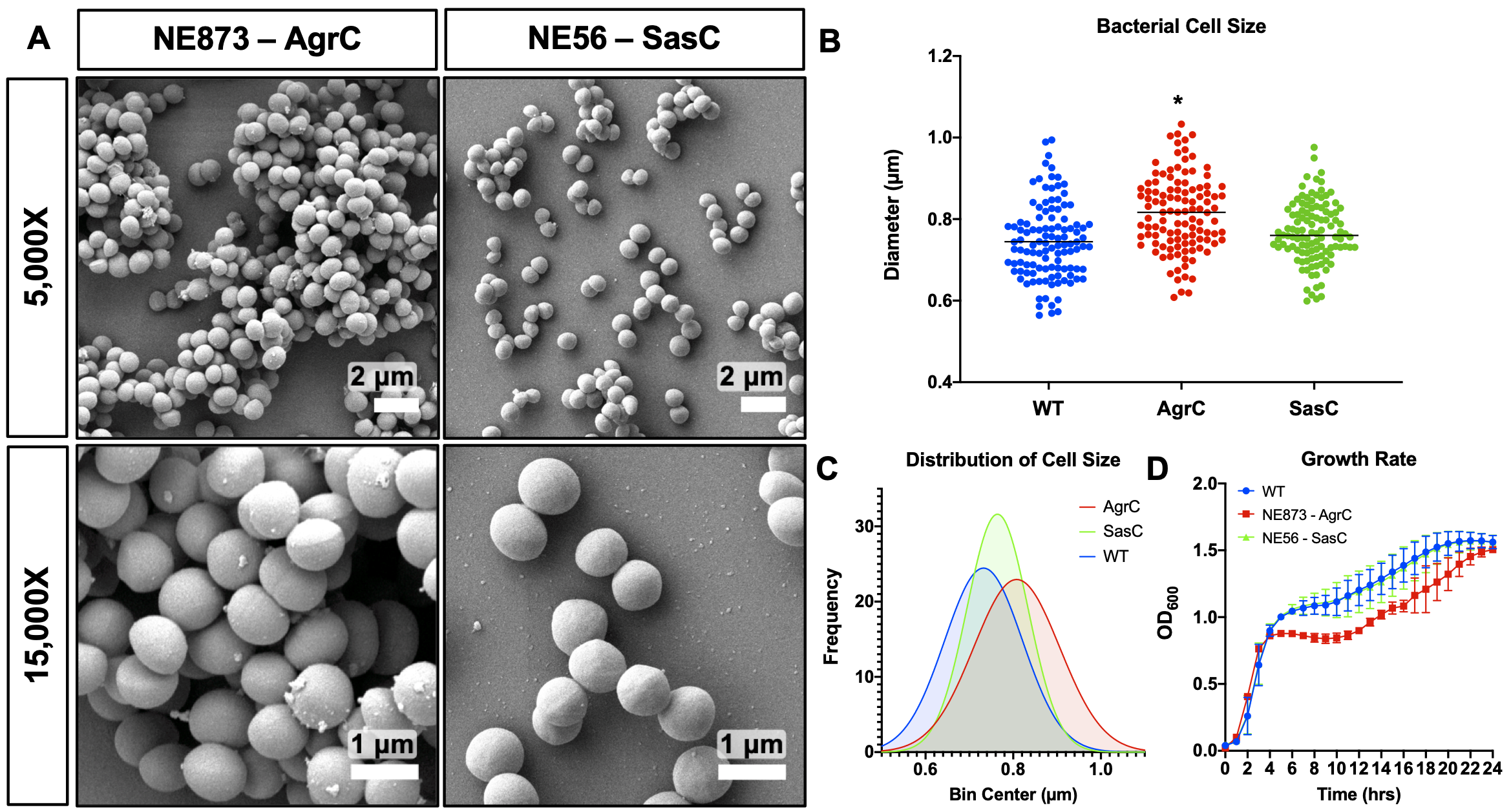

Supplement: S1 Fig — Cultures of NE873-AgrC and NE56-SasC were assessed by SEM (A) and optical density at 600 nm (B) as described in Fig 2. The SEM results confirmed the predicted cell clumping phenotype of the agrC mutant, and also demonstrated that these bacteria are slightly larger than WT cells (B, *p<0.05, by one-way ANOVA with Tukey’s post-hoc for multiple comparisons). Interestingly, their growth rate is significantly hindered during stationary phase from 5–21 hours of incubation (D, two-way ANOVA with Sidak’s post-hoc for multiple comparisons). Taken together, the identification of NE873-AgrC in the genetic screen may be a false-positive because of its increased cell size and hindered growth. In contrast, SEM revealed that sasC mutants are similar to WT in size (B, C) and growth rate (D). (TIFF) [file ppat.1008988.s001.tiff]

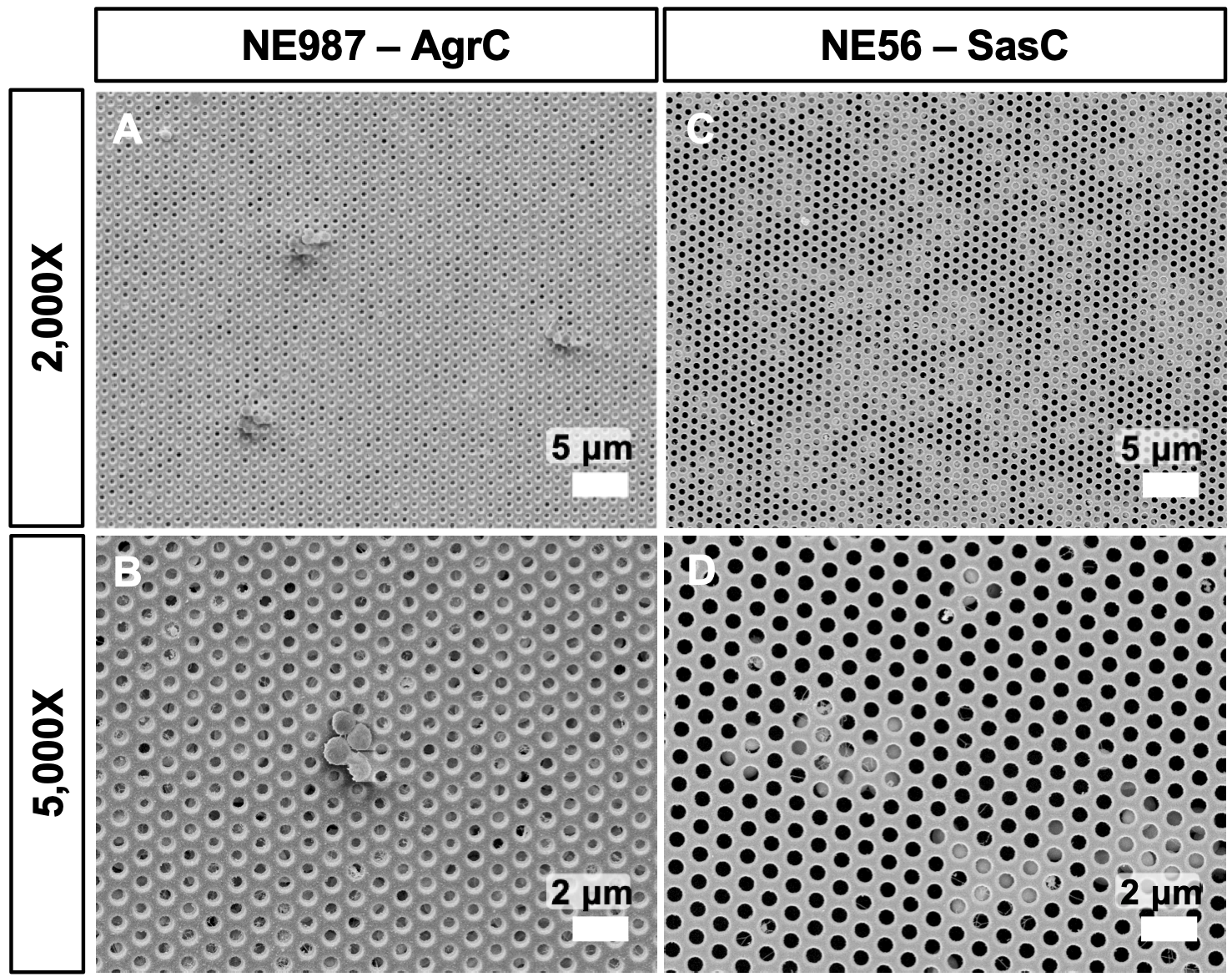

Supplement: S2 Fig — Cultures of NE873-AgrC and NE56-SasC were evaluated for nanopore propagation using methods described in Fig 3. Representative SEM micrographs (n = 4 independent experiments) confirm agrC mutant propagation through 0.5 μm nanopores (A, B), thereby confirming that this mutant strain was identified as a false-positive in the pooled genetic screen. In contrast, representative SEM micrographs (n = 4 independent experiments) of the sasC transposon mutant confirm that it is incapable of propagation through the nanopores (C, D). (TIFF) [file ppat.1008988.s002.tiff]

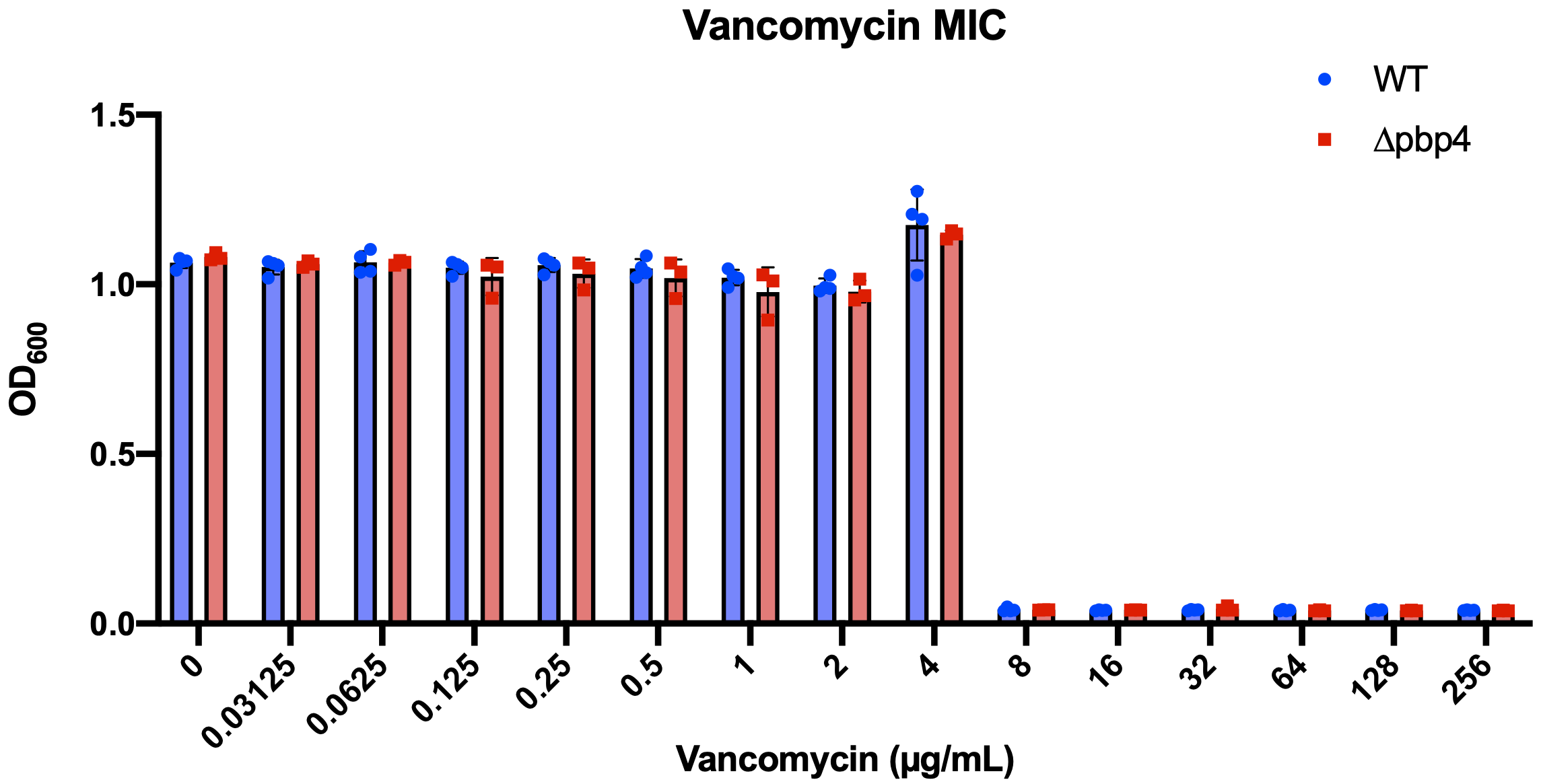

Supplement: S3 Fig — Liquid cultures (n = 4) of WT and Δpbp4 USA300 were grown in the indicated concentration of vancomycin overnight at 37°C with shaking, and the optical densities of the cultures at 600 nm are presented as the mean +/- SD. No differences between strains were observed at any concentration (two-way ANOVA, with Sidaks’s post-hoc for multiple comparisons). The resultant MIC of both strains, simply measured by OD600, is approximately 8 μg/mL. (TIFF) [file ppat.1008988.s003.tiff]

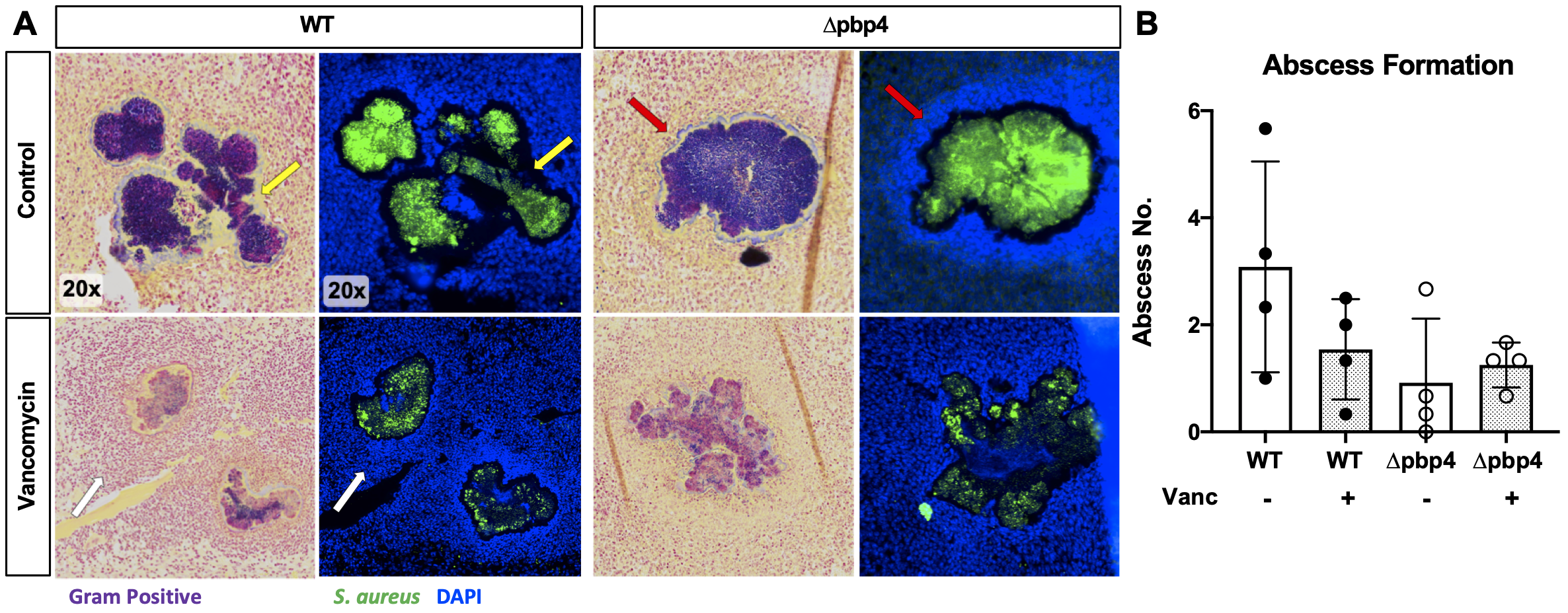

Supplement: S4 Fig — Parallel histology sections of tibiae from the four infection groups were processed for Brown-Brenn staining and anti-S. aureus IHC as described in Fig 7, and representative micrographs of SACs in the tibial marrow space are shown at 20x. Note the overlapping Gram stained bacteria (purple) with the immunostaining (green). All infection groups show robust SAC formation, where S. aureus are located at the center of an abscess initially surrounded by a fibrous pseudo-capsule shown yellow in BB stain and as a black ring in IF staining (yellow arrow). Next, surrounding the fibrous pseudo-capsule is a ring of live and dead immune cells (red arrow), followed by living immune cells unable to penetrate the abscess structure (white arrow). Abscesses per tibia were quantified by averaging abscess number over 3 histological levels for 4 biological replicates (B). While there appears to be a trend toward decreased abscess number with vancomycin treatment and Δpbp4, no significant differences between groups were found (one-way ANOVA with Tukey’s post-hoc for multiple comparisons). (TIFF) [file ppat.1008988.s004.tiff]

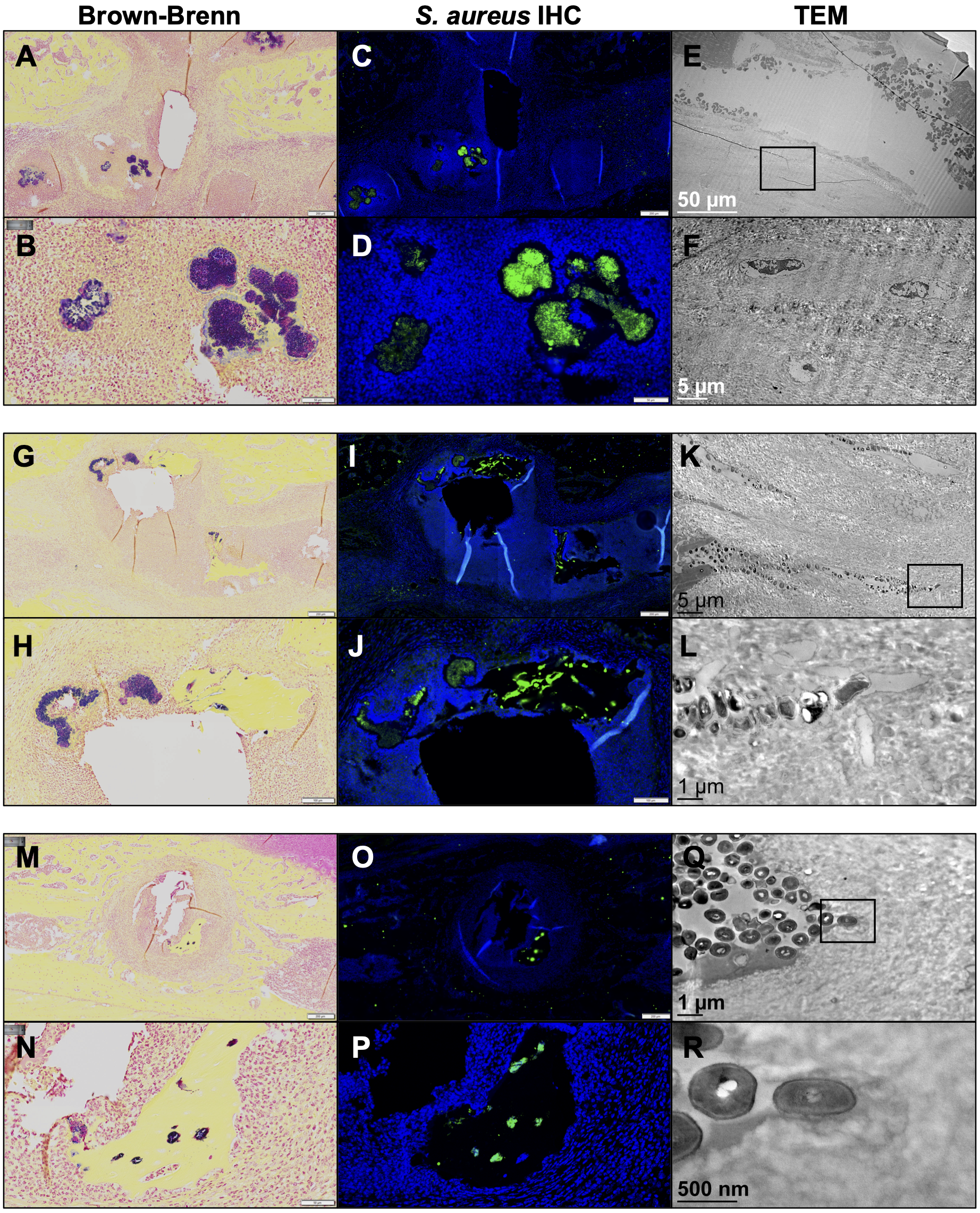

Supplement: S5 Fig — Infected tibiae were processed for histology and sections were stained with Brown-Brenn (A, B, H, H, M, N), anti-S. aureus IHC (C, D, I, J, O, P) and processed for TEM “pop-off” (E, F, K, L, Q, R). TEM was imaging was blinded to sample group assignment. Representative images from all 3 biological replicates from this infection group are shown. (TIFF) [file ppat.1008988.s005.tiff]

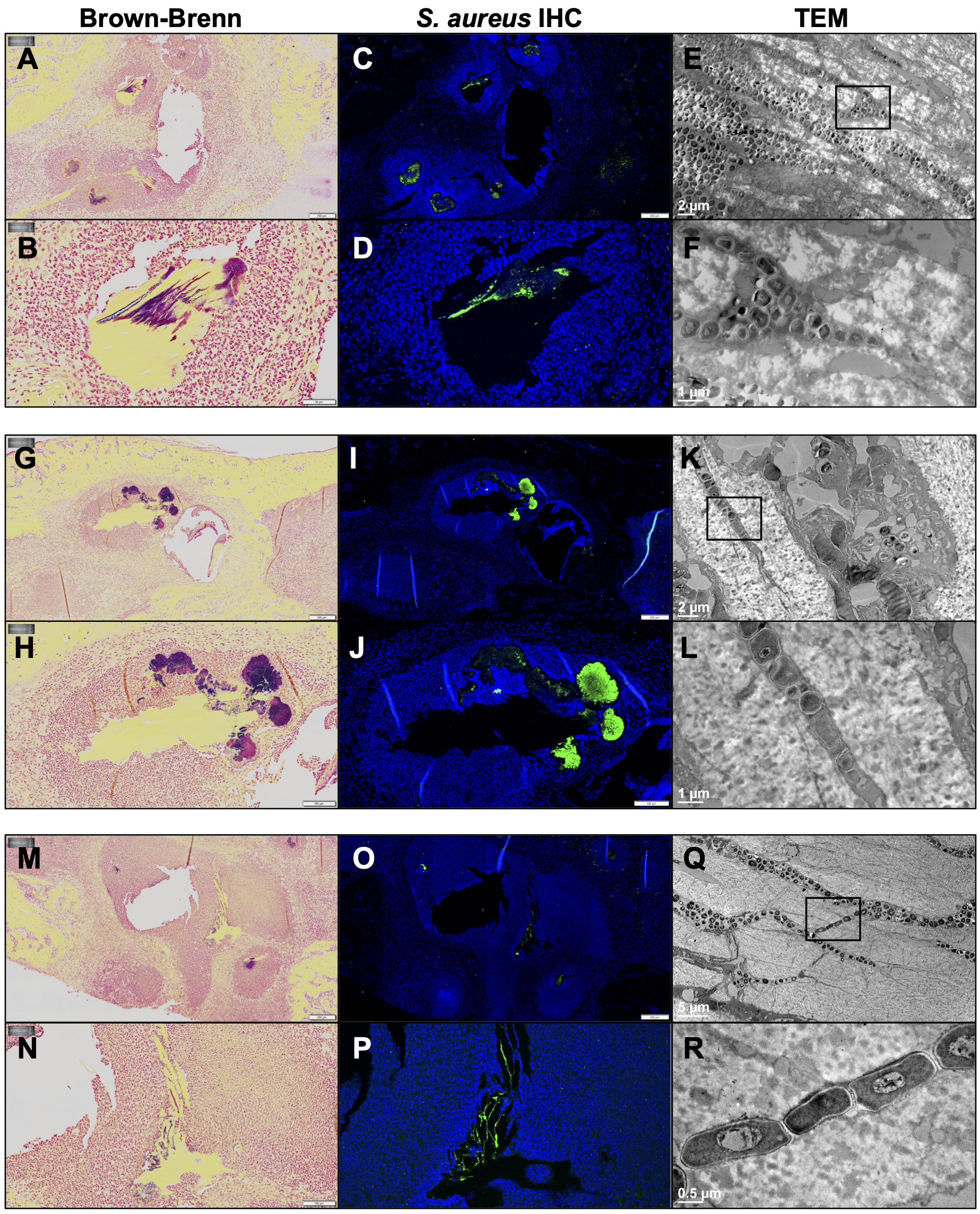

Supplement: S6 Fig — Infected tibiae were processed for histology and sections were stained with Brown-Brenn (A, B, H, H, M, N), anti-S. aureus IHC (C, D, I, J, O, P) and processed for TEM “pop-off” (E, F, K, L, Q, R). TEM was imaging was blinded to sample group assignment. Representative images from all 3 biological replicates from this infection group are shown. (TIFF) [file ppat.1008988.s006.tiff]

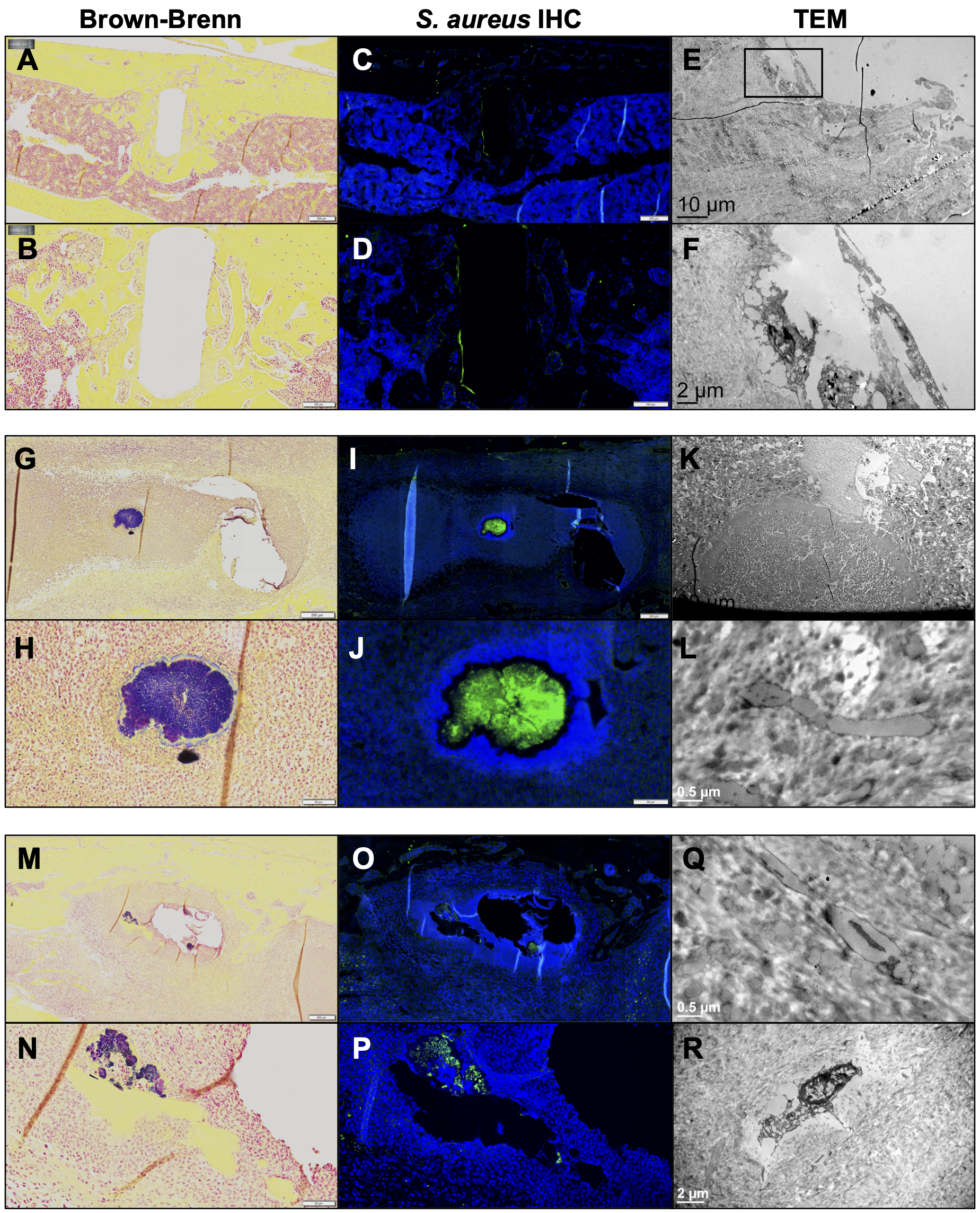

Supplement: S7 Fig — Infected tibiae were processed for histology and sections were stained with Brown-Brenn (A, B, H, H, M, N), anti-S. aureus IHC (C, D, I, J, O, P) and processed for TEM “pop-off” (E, F, K, L, Q, R). TEM was imaging was blinded to sample group assignment. Representative images from all 3 biological replicates from this infection group are shown. (TIFF) [file ppat.1008988.s007.tiff]

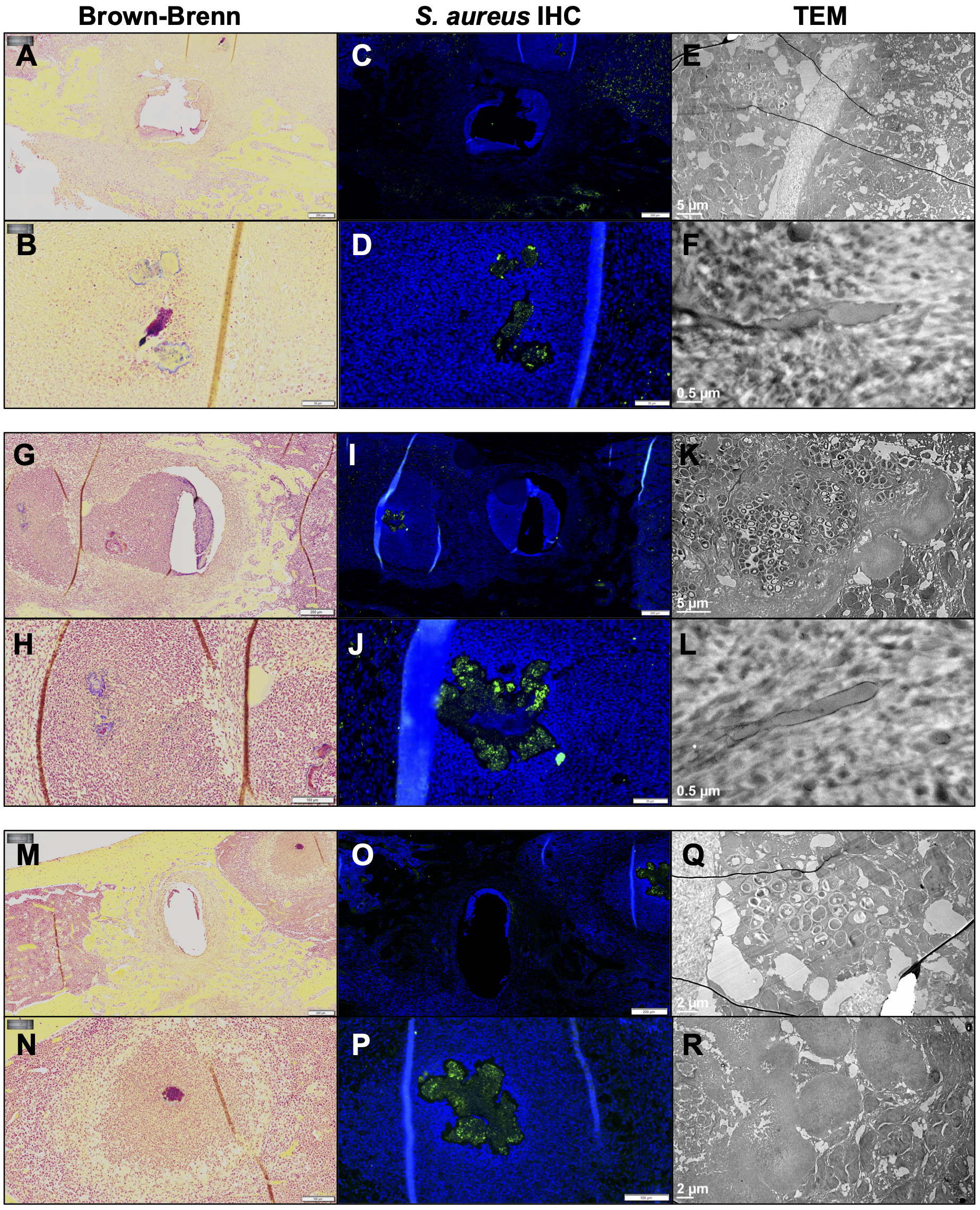

Supplement: S8 Fig — Infected tibiae were processed for histology and sections were stained with Brown-Brenn (A, B, H, H, M, N), anti-S. aureus IHC (C, D, I, J, O, P) and processed for TEM “pop-off” (E, F, K, L, Q, R). TEM was imaging was blinded to sample group assignment. Representative images from all 3 biological replicates from this infection group are shown. (TIFF) [file ppat.1008988.s008.tiff]

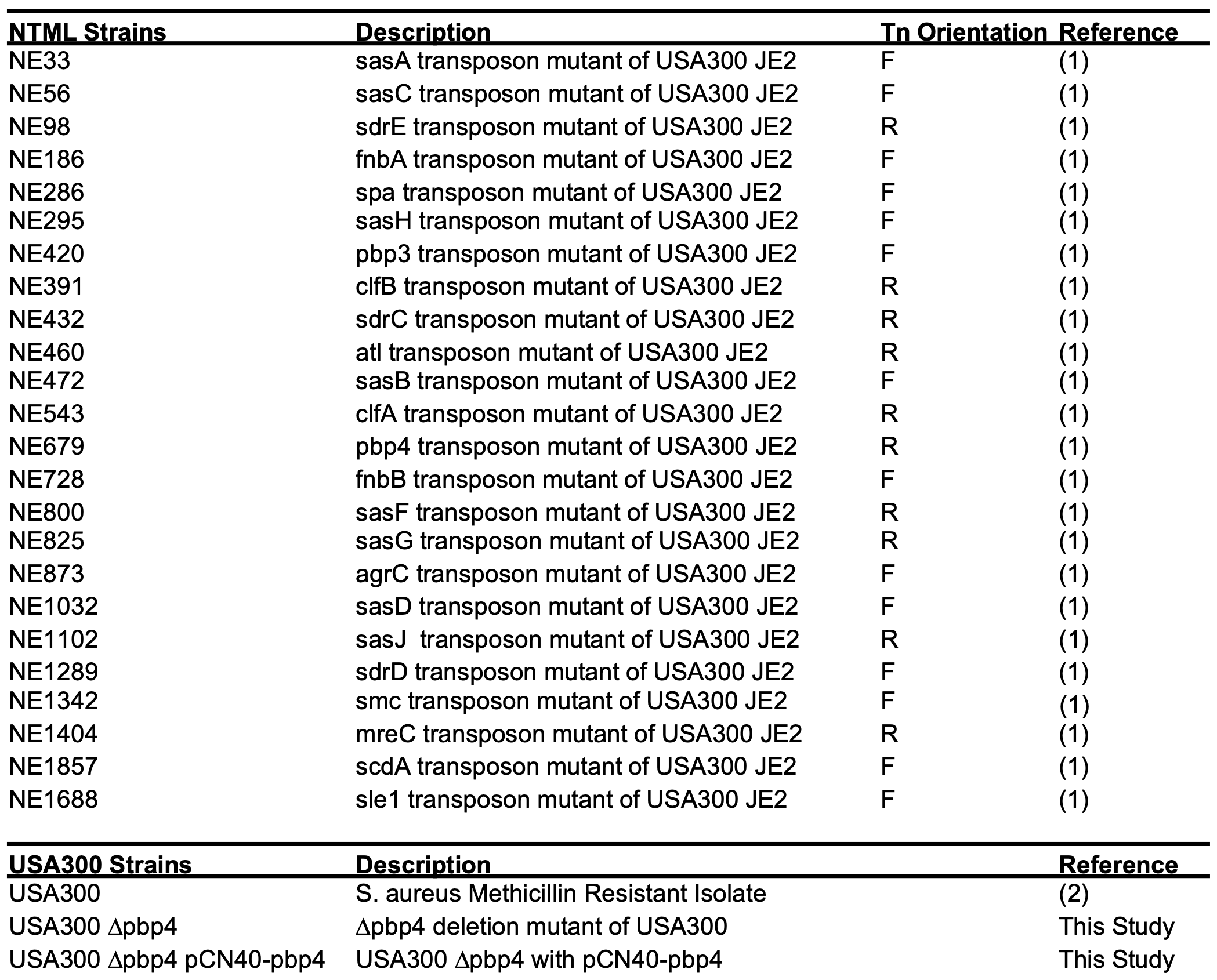

Supplement: S1 Table — References: (1) Fey PD, et al. 2013. MBio 4:e00537-12. (2) Diep BA, et al. 2006. The Lancet 367:731–739. (TIFF) [file ppat.1008988.s009.tiff]

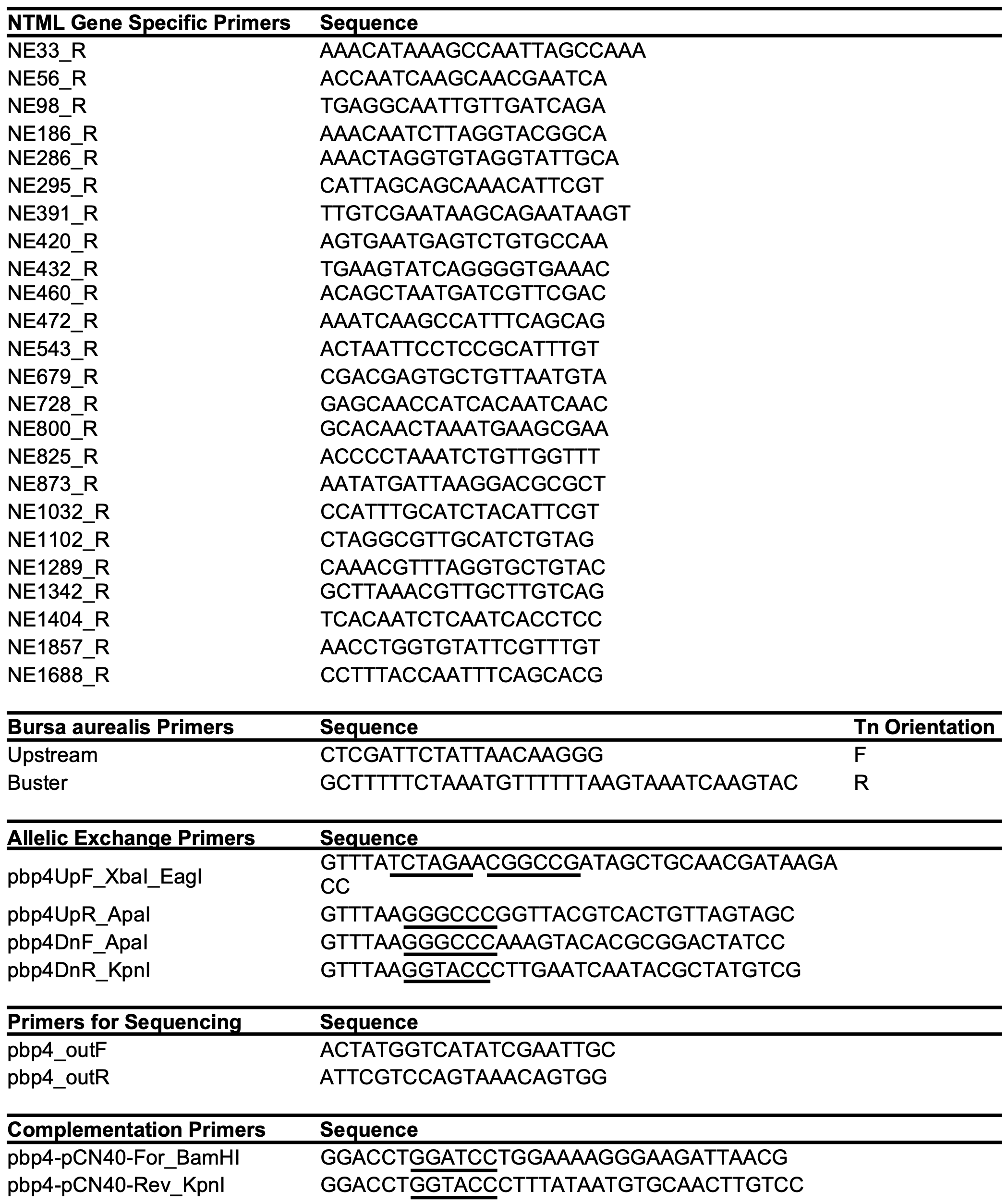

Supplement: S2 Table — (TIFF) [file ppat.1008988.s010.tiff]
